# Performance of risk scores in predicting major bleeding in left ventricular assist device recipients: a comparative external validation

**DOI:** 10.1016/j.rpth.2024.102437

**Published:** 2024-05-06

**Authors:** S.F.B. van der Horst, Y. de Jong, N. van Rein, J.W. Jukema, M. Palmen, E. Janssen, E.F. Bonneville, F.A. Klok, M.V. Huisman, L.F. Tops, P.L. den Exter

**Affiliations:** 1 Department of Medicine—Thrombosis and Hemostasis, Leiden University Medical Center, Leiden, The Netherlands; 2 Department of Clinical Epidemiology, Leiden University Medical Center, Leiden, The Netherlands; 3 Department of Clinical Pharmacy and Toxicology, Leiden University Medical Center, Leiden, The Netherlands; 4 Department of Cardiology, Leiden University Medical Center, Leiden, The Netherlands; 5 Department of Thoracic Surgery, Leiden University Medical Center, Leiden, The Netherlands; 6 Department of Biomedical Data Sciences, Leiden University Medical Center, Leiden, The Netherlands

**Keywords:** anticoagulants, clinical decision rules, heart-assist devices, hemorrhage, validation studies as topic

## Abstract

**Background:**

Implantation of a left ventricular assist device (LVAD) is a crucial therapeutic option for selected end-stage heart failure patients. However, major bleeding (MB) complications postimplantation are a significant concern.

**Objectives:**

We evaluated current risk scores’ predictive accuracy for MB in LVAD recipients.

**Methods:**

We conducted an observational, single-center study of LVAD recipients (HeartWare or HeartMate-3, November 2010-December 2022) in the Netherlands. The primary outcome was the first post-LVAD MB (according to the International Society on Thrombosis and Haemostasis [ISTH] and Interagency Registry for Mechanically Assisted Circulatory Support [INTERMACS], and INTERMACS combined with intracranial bleeding [INTERMACS+] criteria). Mortality prior to MB was considered a competing event. Discrimination (C-statistic) and calibration were evaluated for the Hypertension, Abnormal Renal/Liver Function, Stroke, Bleeding History or Predisposition, Labile INR, Elderly, Drugs/Alcohol Concomitantly score, Hepatic or Renal Disease, Ethanol Abuse, Malignancy, Older Age, Reduced Platelet Count or Function, Re-Bleeding, Hypertension, Anemia, Genetic Factors, Excessive Fall Risk and Stroke score, Anticoagulation and Risk Factors in Atrial Fibrillation score, Outpatient Bleeding Risk Index, venous thromboembolism score, atrial fibrillation score, and Utah Bleeding Risk Score (UBRS).

**Results:**

One hundred four patients were included (median age, 64 years; female, 20.2%; HeartWare, 90.4%; HeartMate-3, 9.6%). The cumulative MB incidence was 75.7% (95% CI 65.5%-85.9%) by ISTH and INTERMACS+ criteria and 67.0% (95% CI 56.0%-78.0%) per INTERMACS criteria over a median event-free follow-up time of 1916 days (range, 59-4521). All scores had poor discriminative ability on their intended prediction timeframe. Cumulative area under the receiving operator characteristic curve ranged from 0.49 (95% CI 0.35-0.63, venous thromboembolism-BLEED) to 0.56 (95% CI 0.47-0.65, UBRS) according to ISTH and INTERMACS+ criteria and from 0.48 (95% CI 0.40-0.56, Anticoagulation and Risk Factors in Atrial Fibrillation) to 0.56 (95% CI 0.47-0.65, UBRS) per INTERMACS criteria. All models showed poor calibration, largely underestimating MB risk.

**Conclusion:**

Current bleeding risk scores exhibit inadequate predictive accuracy for LVAD recipients. There is a need for an accurate risk score to identify LVAD patients at high risk of MB who may benefit from patient-tailored antithrombotic therapy.

## Essentials

• Left ventricular assist device (LVAD) recipients are at high risk of major bleeding (MB).
• We assessed current risk scores’ predictive accuracy for MB in a single-center Dutch LVAD cohort.
• None of the evaluated scores was able to accurately predict MB.
• There is a need for an accurate risk score to reliably identify LVAD recipients at high MB risk.

## 1 Introduction

Left ventricular assist device (LVAD) implantation has emerged as a pivotal circulatory support strategy for selected patients with advanced systolic heart failure, either as a bridge to transplant or destination therapy for those ineligible for heart transplantation [1]. To mitigate thromboembolic risk, in particular pump thrombosis and ischemic stroke, LVAD patients require long-term dual antithrombotic therapy. Current international guidelines advise a combination of antiplatelet therapy with a vitamin K antagonist (VKA), targeting an international normalized ratio (INR) in the range of 2.0 to 3.0 [2]. However, this intensive anticoagulant management in combination with post-LVAD hemostatic changes, particularly acquired von Willebrand syndrome and platelet dysfunction, pose LVAD recipients at high risk of bleeding [3]. Based on data from the Society of Thoracic Surgeons Interagency Registry for Mechanically Assisted Circulatory Support (INTERMACS) involving 13,945 continuous-flow LVAD patients, major bleeding (MB) is a prevalent adverse event following LVAD implantation, especially in the first 90 days (event rate ≤ 90 days: 122 MB/100 patient-years [PY] compared with 25/100 PY thereafter) [4].

While the newer generation LVAD HeartMate-3 (Abbott) demonstrates enhanced thromboembolic outcomes compared with HeartMate-2 and HeartWare (Medtronic), MB remains a concern irrespective of the implanted device [5,6]. This high bleeding incidence necessitates patient-tailored anticoagulant care. To improve clinical decision making regarding antithrombotic strategies, an accurate risk assessment tool is needed. To date, the applicability of commonly used risk scores for MB in the atrial fibrillation (AF) population (eg, the Hypertension, Abnormal Renal/Liver Function, Stroke, Bleeding History or Predisposition, Labile INR, Elderly, Drugs/Alcohol Concomitantly [HAS-BLED] score and Hepatic or Renal Disease, Ethanol Abuse, Malignancy, Older Age, Reduced Platelet Count or Function, Re-Bleeding, Hypertension, Anemia, Genetic Factors, Excessive Fall Risk and Stroke [HEMORR_2_HAGES] score) has only scarcely been investigated in LVAD patients, and the limited results regarding predictive performance are contradictory [[7], [8], [9]]. The Utah Bleeding Risk Score (UBRS) is the only risk score specifically developed for LVAD recipients, aiming to predict gastrointestinal bleeding (GIB) [10]. However, the discriminative ability upon external validation was disappointing (area under the receiving operator characteristic curve [AUC], 0.52; 95% CI 0.42-0.62) [11].

We performed an observational cohort study with the aim of evaluating and comparing the predictive accuracy of current risk scores for MB in LVAD recipients. Our head-to-head comparative validation included 7 risk scores: HAS-BLED, HEMORR_2_HAGES, Anticoagulation and Risk Factors in Atrial Fibrillation (ATRIA), venous thromboembolism (VTE)-BLEED, atrial fibrillation (AF)-BLEED, Outpatient Bleeding Risk Index (OBRI), and UBRS [10,[12], [13], [14], [15], [16], [17]].

## 2 Methods

We adhered to the Strengthening the Reporting of Observational Studies in Epidemiology (STROBE) and Transparent Reporting of a multivariable prediction model for Individual Prognosis Or Diagnosis (TRIPOD) statements (Supplementary Material, “STROBE Statement” and “TRIPOD Checklist”) [18].

### 2.1 Ethical considerations

The study protocol was approved by the local cardiopulmonary scientific committee of the Leiden University Medical Center (LUMC), which deemed the research outside the scope of the Medical Research Involving Human Subjects Act (in Dutch: Wet medisch-wetenschappelijk onderzoek met mensen, WMO), thereby obviating the need for formal approval and informed consent.

### 2.2 Study design and participants

We conducted a single-center cohort study including all consecutive adult patients (aged ≥18 years) undergoing implantation of either a HeartWare-Medtronic or HeartMate-3 LVAD at the LUMC, the Netherlands, between November 2010 and December 2022. The dataset originated from the LUMC LVAD database (extracted from electronic health records) and was augmented with additional variables retrieved from electronic health records through comprehensive review. Notably, HeartWare was the only LVAD that has been implanted in LUMC until June 2021. From October 2021, LVAD implantation was solely performed with HeartMate-3. Follow-up extended from LVAD implantation until the first MB event, death, transfer to another hospital, or the end of the study period (May 1, 2023), whichever occurred first.

### 2.3 Baseline characteristics

We collected data on demographics (age and biological sex) and clinical information (medical history, clinical diagnosis necessitating LVAD implantation, weight, length, blood pressure, right ventricular function, mean pulmonary artery pressure [MPAP], laboratory findings, and details regarding the LVAD surgery). Baseline was defined as the most recent recording prior to LVAD implantation.

### 2.4 Outcome

The primary outcome was the time from implantation to first MB during the follow-up period. To ensure uniformity in comparing model performance with a clinically relevant outcome, we applied consistent outcome definitions across all validated models. MB was defined according to INTERMACS criteria (Mechanical Circulatory Support—Academic Research Consortium [MCS-ARC] adverse event definitions—MCS-ARC bleeding type 3, 4, or 5), INTERMACS+ criteria (MCS-ARC bleeding type 3, 4, or 5, combined with intracranial hemorrhage), and International Society on Thrombosis and Haemostasis (ISTH) criteria for MB in nonsurgical patients (Supplementary Table S1) [19,20]. We recorded the first MB event post-LVAD implantation, considering each of 3 definitions separately. In cases where MB occurred according to 1 or 2 definitions (ISTH, INTERMACS, or INTERMACS+) but not according to all 3, we assessed whether subsequent MB events meeting the alternative definition(s) occurred until the study’s end date. MB complications directly related to LVAD implantation surgery were not considered. Surgical-related MB was defined as any MB within the 48-hour postoperative window following LVAD implantation or an MB occurring outside the 48-hour window but related to an MB within the 48-hour window.

### 2.5 Validated bleeding risk scores and predictor definition

The predictive performance of the following bleeding risk scores was evaluated: HAS-BLED, HEMORR_2_HAGES, ATRIA, VTE-BLEED, AF-BLEED, OBRI, and UBRS (Supplementary Table S2) [10,[12], [13], [14], [15], [16], [17]]. These risk scores were selected based on the presence of extensive validation studies and their routine clinical application. We aligned our predictor definitions with those specified in the development studies, ensuring validation of the models as originally intended. The predictors included in each risk score, along with the predictor definitions, are listed in Supplementary Table S3. The scores were calculated using pre-LVAD implantation patient data. Outcome evaluation was performed without awareness of the risk score sums.

### 2.6 Statistical analyses

For each patient, risk scores and the predicted probability of experiencing an MB were calculated. In the case of missing data (prior bleeding: *n* = 5 [4.8%] and MPAP: *n* = 5 [4.8%]), a score of zero was assigned for that variable. All risk scores provided risk categories (eg, low, intermediate, or high risk). Given the absence of formulas for the risk scores included, we calculated the scores for each patient by summing the point scores for predictors, which were then linked to their corresponding predicted probabilities. These predicted probabilities represent MB event rates or cumulative incidences associated with each risk score, as reported in the original articles (Supplementary Table S4). When risk scores provided event rates rather than cumulative incidences, we approximated the cumulative incidence (detailed in Supplementary Material, “Strategy to convert event rates (EVR) to approximated cumulative incidences”) [21,22].

For each definition (ISTH, INTERMACS, and INTERMACS+), MB outcomes were reported separately. Competing risk analyses were conducted to calculate the cumulative incidence of MB using the Aalen–Johansen estimator of the cumulative incidence function. Death before reaching the defined MB outcome was considered a competing event. Patients not experiencing MB and still alive at the end of follow-up were censored. The median follow-up duration among those event-free (ie, alive and not having experienced an MB) was calculated by the reverse Kaplan–Meier (KM) estimate of the survival function.

Performance of the risk scores in predicting MB was evaluated in terms of their discriminative ability and calibration, accounting for competing risk. Discrimination, measuring the model's capacity to distinguish between individuals who experienced the outcome of interest and those who did not, was evaluated using the cumulative AUC (AUC_t_). Right-censoring was addressed by inverse-probability-of-censoring weighting. An AUC_t_ of 1 indicates perfect discrimination, 0 perfect inverse discrimination, and 0.5 random chance (akin to flipping a coin). Generally, an AUC_t_ or C-statistic of <0.7 is considered poor, ≥0.7 moderate, ≥0.8 good, and ≥0.9 excellent [23].

Calibration assesses how well model’s predicted probabilities align with observed probabilities, which is a crucial characteristic of any risk score. The nonparametric cumulative incidence estimates for each risk score were used as the observed outcome and were plotted against the corresponding predicted probabilities in order to obtain a calibration plot [24,25]. Additionally, we calculated the observed/expected ratio, calibration intercept (calibration-in-the-large), and calibration slope with 95% CIs for each model.

We validated the scores within their intended timeframe specified in the original articles (eg, HAS-BLED, ATRIA, OBRI 1 year, VTE-BLEED 30 days to 6 months, AF-BLEED 180 days, UBRS 3 years) or within the maximum follow-up period of the development cohort if no specific timeframe was stated (eg, HEMORR_2_HAGES 1000 days). Additionally, we sequentially evaluated C-statistics over time at monthly intervals up to the intended timeframe.

All statistical analyses were conducted using R (version 4.1.2, R Foundation for Statistical Computing) with packages cmprsk, survival, prodlim, riskRegression, and pec.

#### 2.6.1 Sensitivity analyses

We conducted 6 sensitivity analyses. First, we evaluated cumulative MB incidences among device types (HeartWare vs HeartMate-3) and antiplatelet types (acetylsalicylic acid/carbasalate calcium [ASA] vs clopidogrel). However, predictive performance measures could not be performed within these subgroups due to the small group sizes of those implanted with HeartMate-3 and those prescribed ASA.

Second, to address for missing data, we performed a complete case analysis for the primary outcome instead of assigning a score of 0 for missing variables.

Third, to validate the models as initially developed, ie, without considering death as competing event, we applied the previously mentioned statistical techniques with a modification: “mortality without having experienced an MB” was treated as a censoring event instead of a competing risk. Cumulative MB incidences were calculated by taking 1 minus the KM (1 – KM) estimate. For assessing discrimination and calibration, we followed the same statistical procedures as described earlier, with the only difference in calibration, where observed probabilities obtained using 1 – KM instead of the nonparametric Aalen–Johansen estimator were plotted against predicted probabilities.

Fourth, discriminative ability was evaluated by the Harrell *C*-index (ranking times-to-event; patients with higher risk scores are expected to have a shorter time-to-event, ie, MB) instead of the AUC_t_; 95% CIs were obtained by bootstrapping the *C*-index (B = 200 bootstrap samples).

Fifth, we extended the postoperative window following LVAD implantation to 14 days instead of 48 hours and only included MB occurring outside of this timeframe to further ensure that all surgical bleeding complications were excluded. Additionally, thrombolysis, alongside mortality, was treated as a competing event, as MB is an expected complication.

Lastly, we aimed to assess correlation between risk scores and MB. To provide insight into MB incidences among risk categories (low, intermediate, or high), we constructed cumulative incidence plots over time for each risk group and tested the difference with Gray’s test (*P* < .05 for significance). Additionally, for each score (discrete and categorical), subdistribution hazard ratios and corresponding 95% CIs were obtained from the univariate Fine–Gray model.

## 3 Results

### 3.1 Patients

The study included 104 patients who underwent HeartWare or HeartMate-3 device implantation between November 9, 2010, and December 21, 2022. Table 1 summarizes the clinical characteristics of the patients. The median age at LVAD implantation was 64.0 years (quartile 1-quartile 3 [Q1-Q3], 58.0-68.0), and 20.2% were women. Heart failure etiologies necessitating LVAD implantation were mostly ischemic cardiomyopathy (*n* = 58; 55.8%) and nonischemic dilated cardiomyopathy (*n* = 35; 33.7%). All patients were in New York Heart Association class IV (53.8%) or III (46.2%), and most patients were in INTERMACS profile 3 (40.4%) at implantation. Of the cohort, 94 patients (90.4%) received the HeartWare device and 10 patients (9.6%) received the HeartMate-3 device, primarily as destination therapy (*n* = 101; 97.1%). None underwent cardiac transplantation or LVAD explantation.

**Table 1:** Clinical characteristics of the included patients.

Characteristics	All (*N* = 104), *n* (%)^a^
**Age at implantation in y**, median (Q1-Q3)	64 (58-68)
**Biological sex, women**	21 (20.2)
**Heart failure etiology**	
Ischemic cardiomyopathy	58 (55.8)
Nonischemic dilative cardiomyopathy	35 (33.7)
Valvular heart disease	6 (5.8)
Congenital heart disease	4 (3.8)
Hypertrophic cardiomyopathy	1 (1.0)
**NYHA class**	
III	48 (46.2)
IV	56 (53.8)
**INTERMACS profile preimplantation**	
Profile 1	4 (3.8)
Profile 2	18 (17.3)
Profile 3	42 (40.4)
Profile 4	28 (26.9)
Profile 5	12 (11.5)
**Risk scores preimplantation**, median (Q1-Q3)	
HAS-BLED	1 (1-2)
HEMORR_2_HAGES	1 (1-2)
ATRIA	1 (0-3)
OBRI	1 (1-2)
VTE-BLEED	3 (1.5-4.5)
AF-BLEED	1.5 (1.5-3)
UBRS	3 (2-4)
**LVAD implanted**	
HeartWare	94 (90.4)
HeartMate-3	10 (9.6)
**Device strategy**	
Destination therapy	101 (97.1)
Rescue therapy	2 (1.9)
Bridge to recovery	1 (1.0)

AF-BLEED, atrial fibrillation-BLEED; ATRIA, Anticoagulation and Risk Factors in Atrial Fibrillation; HAS-BLED, Hypertension, Abnormal Renal/Liver Function, Stroke, Bleeding History or Predisposition, Labile INR, Elderly, Drugs/Alcohol Concomitantly; HEMORR_2_HAGES, Hepatic or Renal Disease, Ethanol Abuse, Malignancy, Older Age, Reduced Platelet Count or Function, Re-Bleeding, Hypertension, Anemia, Genetic Factors, Excessive Fall Risk and Stroke; INTERMACS, Interagency Registry for Mechanically Assisted Circulatory Support; LVAD, left ventricular assist device; NYHA, New York Heart Association; OBRI, Outpatient Bleeding Risk Index; Q1-Q3, quartile 1 to quartile 3; UBRS, Utah Bleeding Risk Score; VTE-BLEED, venous thromboembolism-BLEED.

a Unless otherwise specified.

Follow-up was complete, except for 1 patient who was transferred to another hospital 2 months post-LVAD implantation. The median follow-up time among those event-free was 1916 days (range, 59-4521).

### 3.2 Antithrombotic therapy

Postimplantation, 86 patients (82.7%) were anticoagulated with phenprocoumon, maintaining an INR target range of 2.0 to 3.0. They were concomitantly prescribed clopidogrel 75 mg once daily (*n* = 76; 73.1%), carbasalate calcium 100 mg once daily (*n* = 6; 5.8%), or acetylsalicylic acid 80 mg once daily (*n* = 4; 3.8%). One patient was transferred, and 17 patients died or experienced an MB before phenprocoumon initiation; these patients received unfractionated heparin, either as monotherapy or with antiplatelet therapy.

### 3.3 MB

**Figure 1:**
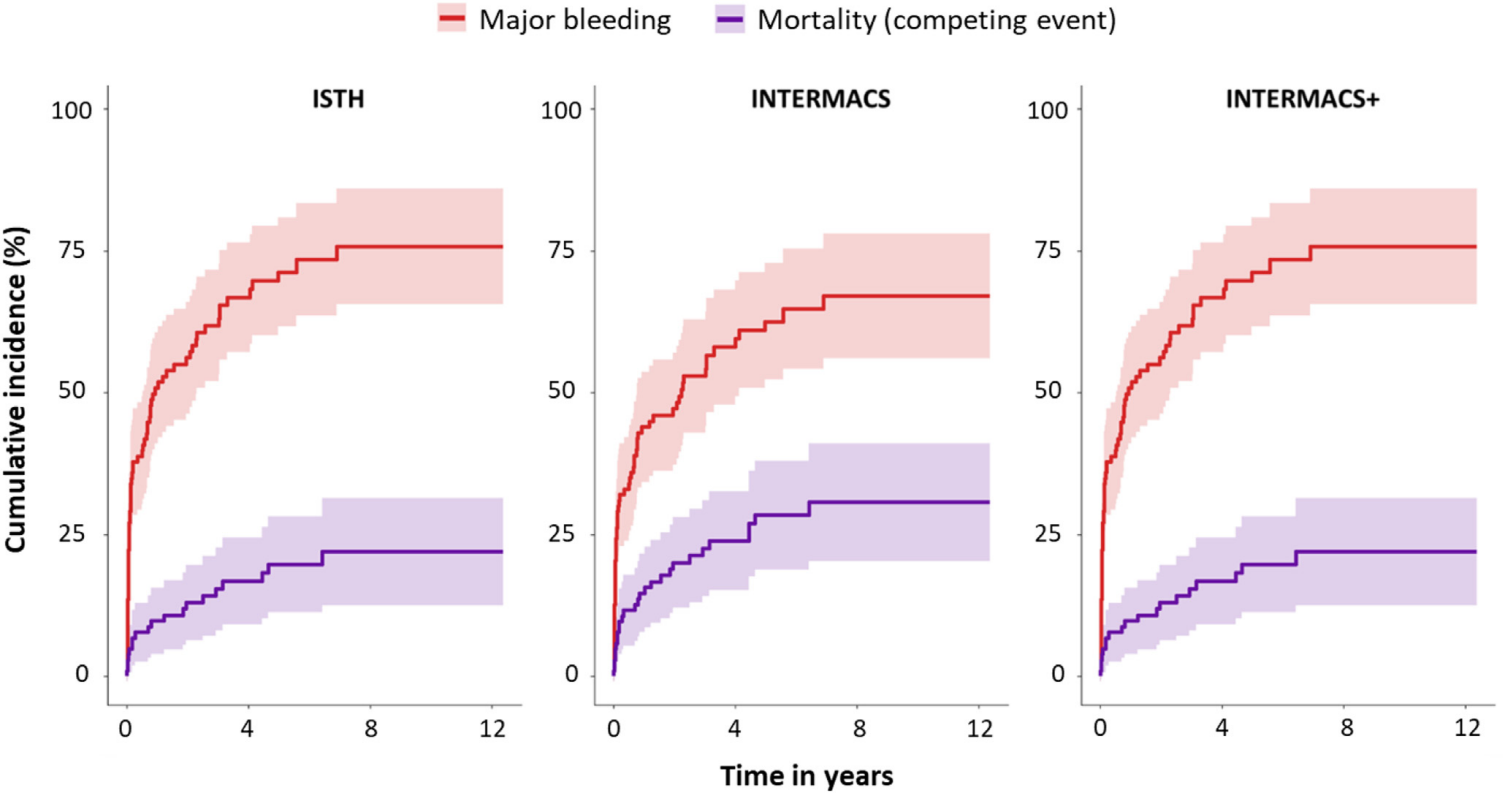
Cumulative incidence of major bleeding and mortality (competing event) over time. Major bleeding was evaluated according to the International Society on Thrombosis and Haemostasis (ISTH), Interagency Registry for Mechanically Assisted Circulatory Support (INTERMACS), and INTERMACS + intracranial bleeding (INTERMACS+) criteria.

Over the entire follow-up duration, the cumulative incidence of MB outside the 48-hour postoperative window was 75.7% (95% CI 65.5%-85.9%) according to ISTH and INTERMACS+ criteria and 67.0% (95% CI 56.0%-78.0%) per INTERMACS criteria (Figure 1 and Supplementary Table S5). The median time to MB was 54 days (ISTH and INTERMACS+: Q1-Q3, 15-404 days; INTERMACS: Q1-Q3, 14.5-453 days). MB occurred in the following sites: gastrointestinal (*n* = 24), mediastinal (*n* = 15, mostly pericardial tamponade; 10 during VKA and 5 during heparin therapy), intracranial (*n* = 11), pleural space (*n* = 5), nasal (*n* = 5), (sub)cutaneous or intramuscular (*n* = 5), driveline exit site (*n* = 3), retroperitoneal (*n* = 2), intraabdominal (*n* = 1), genitourinary (*n* = 1), intraoral (*n* = 1), implantable cardioverter-defibrillator pocket (*n* = 1), and hemorrhagic shock of unknown etiology leading to death (*n* = 1). Four of the MB occurred after intensified anticoagulant therapy (higher dosage of unfractionated heparin or adjustment of INR target range to 3.0-3.5) and 4 MB events were related to alteplase administration in the context of pump thrombosis. In total, 11 patients (10.7%) experienced a fatal bleeding event, of which 1 postthrombolysis for pump thrombosis.

The cumulative incidence of death as competing event was 22.1% (95% CI 12.6%-31.6%) when evaluating MB per ISTH/INTERMACS+ criteria and 30.8% (95% CI 20.3%-41.2%) per INTERMACS criteria (Figure 1 and Supplementary Table S5).

### 3.4 Predictive performance bleeding risk scores

Differences in the distribution of predictors within the derivation cohorts as compared with the present LVAD cohort are summarized in Supplementary Table S6. Fewer LVAD patients met the criteria for hypertension and were assigned points for the age criterion as compared with AF cohorts. Conversely, a higher proportion of LVAD patients were assigned points for prior bleeding events, renal insufficiency, and anemia. The distribution of scores and risk categories are visualized in Supplementary Figure S1. According to most risk scores, the majority of patients were at low risk for bleeding (HAS-BLED score < 2, HEMORR_2_HAGES score < 2, ATRIA score < 4, and AF-BLEED score < 4). Per OBRI and UBRS, most patients had an intermediate risk (OBRI score 1-2 and UBRS score 2-4), while the majority had a high bleeding risk according to the VTE-BLEED score (score ≥ 2).

#### 3.4.1 Discrimination

**Figure 2:**
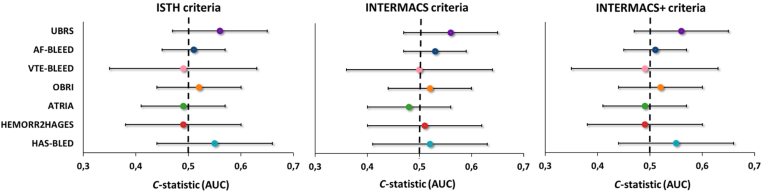
Discriminative ability of the risk scores for major bleeding, with mortality as competing event, according to the International Society on Thrombosis and Haemostasis (ISTH), Interagency Registry for Mechanically Assisted Circulatory Support (INTERMACS), and INTERMACS + intracranial bleeding (INTERMACS+) criteria. Assessment of the cumulative area under the curve (AUCt) was performed on the intended timeframes of the scores (Hypertension, Abnormal Renal/Liver Function, Stroke, Bleeding History or Predisposition, Labile INR, Elderly, Drugs/Alcohol Concomitantly [HAS-BLED], Anticoagulation and Risk Factors in Atrial Fibrillation [ATRIA], Outpatient Bleeding Risk Index [OBRI] 1 year, venous thromboembolism [VTE]-BLEED 30 days to 6 months, atrial fibrillation [AF]-BLEED 180 days, and Utah Bleeding Risk Score [UBRS] 3 years).

**Figure 3:**
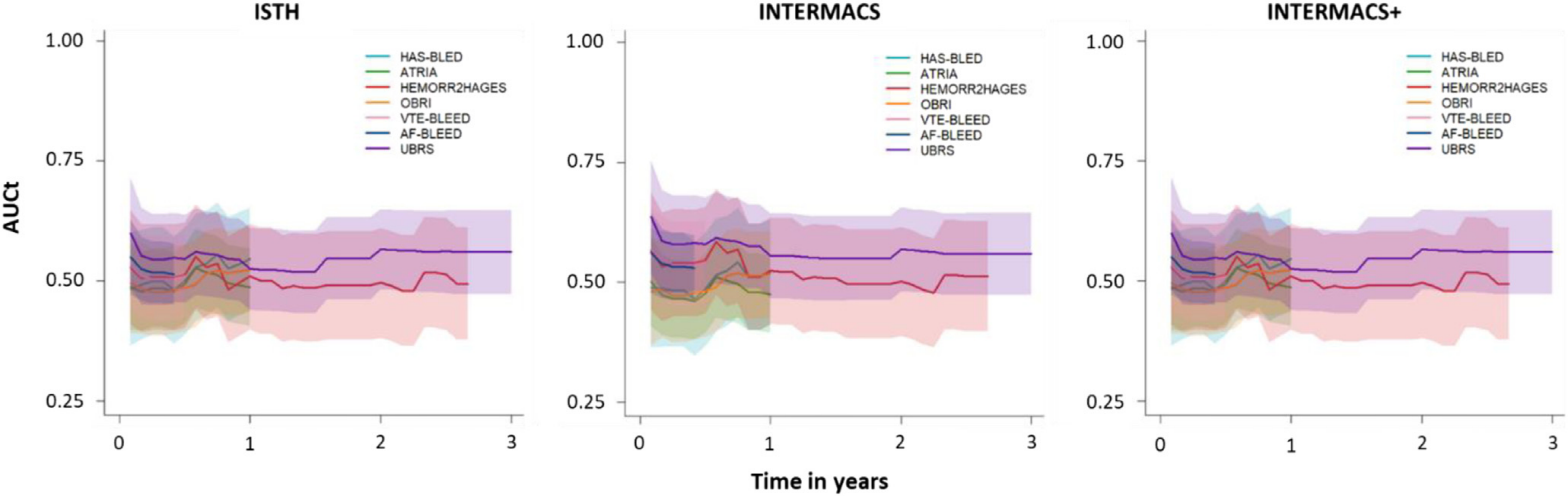
Visualization of the discriminative ability (cumulative area under the curve [AUCt] with 95% CIs) of each risk score for major bleeding over time up to the intended timeframe, with mortality as competing event. Major bleeding was evaluated according to the International Society on Thrombosis and Haemostasis (ISTH), Interagency Registry for Mechanically Assisted Circulatory Support (INTERMACS), and INTERMACS + intracranial bleeding (INTERMACS+) criteria. AF-BLEED, atrial fibrillation-BLEED; ATRIA, Anticoagulation and Risk Factors in Atrial Fibrillation; HAS-BLED, Hypertension, Abnormal Renal/Liver Function, Stroke, Bleeding History or Predisposition, Labile INR, Elderly, Drugs/Alcohol Concomitantly; HEMORR_2_HAGES, Hepatic or Renal Disease, Ethanol Abuse, Malignancy, Older Age, Reduced Platelet Count or Function, Re-Bleeding, Hypertension, Anemia, Genetic Factors, Excessive Fall Risk and Stroke; OBRI, Outpatient Bleeding Risk Index; UBRS, Utah Bleeding Risk Score; VTE-BLEED, venous thromboembolism-BLEED.

The discriminative ability of the bleeding risk scores within the prespecified timeframe according to the AUC_t_ is summarized in Table 2 and Figure 2. All risk scores showed poor discrimination, with AUC_t_ ranging from 0.49 (95% CI 0.35-0.63) to 0.56 (95% CI 0.47-0.65) per ISTH/INTERMACS+ criteria and from 0.48 (95% CI 0.40-0.56) to 0.56 (95% CI 0.47-0.65) per INTERMACS criteria. AUC_t_ remained stable over time for all risk scores (Figure 3).

**Table 2:** Discriminative ability of the risk scores for predicting major bleeding on their intended timeframe.

Risk score	Timeframe	AUCt (95% CI)		
		ISTH	INTERMACS	INTERMACS+
HAS-BLED	1 y	0.55 (0.44-0.65)	0.52 (0.41-0.63)	0.55 (0.44-0.65)
HEMORR_2_HAGES	1000 d	0.49 (0.38-0.61)	0.51 (0.40-0.63)	0.49 (0.38-0.61)
ATRIA	1 y	0.49 (0.41-0.57)	0.48 (0.40-0.56)	0.49 (0.41-0.57)
OBRI	1 y	0.52 (0.44-0.61)	0.52 (0.43-0.61)	0.52 (0.44-0.61)
VTE-BLEED	30 d to 6 mo	0.49 (0.35-0.63)	0.50 (0.34-0.65)	0.49 (0.35-0.63)
AF-BLEED	180 d	0.51 (0.45-0.58)	0.53 (0.46-0.60)	0.51 (0.45-0.58)
UBRS	3 y	0.56 (0.47-0.65)	0.56 (0.47-0.65)	0.56 (0.47-0.65)

Major bleeding, with mortality as competing event, was evaluated according to the International Society on Thrombosis and Haemostasis (ISTH), Interagency Registry for Mechanically Assisted Circulatory Support (INTERMACS), and INTERMACS + intracranial bleeding (INTERMACS+) criteria.

AF-BLEED, atrial fibrillation-BLEED; ATRIA, Anticoagulation and Risk Factors in Atrial Fibrillation; AUCt, cumulative area under the curve; HAS-BLED, Hypertension, Abnormal Renal/Liver Function, Stroke, Bleeding History or Predisposition, Labile INR, Elderly, Drugs/Alcohol Concomitantly; HEMORR_2_HAGES, Hepatic or Renal Disease, Ethanol Abuse, Malignancy, Older Age, Reduced Platelet Count or Function, Re-Bleeding, Hypertension, Anemia, Genetic Factors, Excessive Fall Risk and Stroke; INTERMACS, Interagency Registry for Mechanically Assisted Circulatory Support; INTERMACS+, INTERMACS combined with intracranial bleeding; ISTH, International Society on Thrombosis and Haemostasis; OBRI, Outpatient Bleeding Risk Index; UBRS, Utah Bleeding Risk Score; VTE-BLEED, venous thromboembolism-BLEED.

#### 3.4.2 Calibration

**Figure 4:**
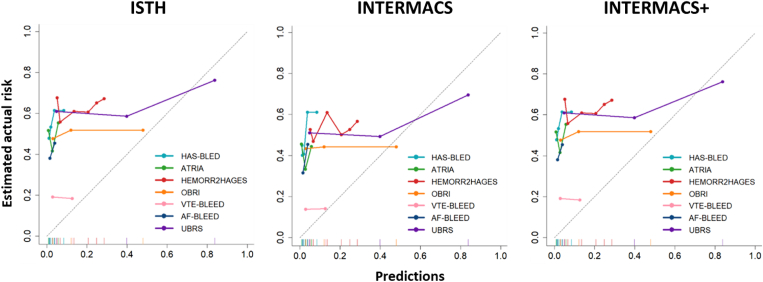
Calibration plots showing predicted and estimated actual risks of major bleeding, with mortality as competing event, on the intended timeframes of the risk scores (Hypertension, Abnormal Renal/Liver Function, Stroke, Bleeding History or Predisposition, Labile INR, Elderly, Drugs/Alcohol Concomitantly [HAS-BLED], Anticoagulation and Risk Factors in Atrial Fibrillation [ATRIA], Outpatient Bleeding Risk Index [OBRI] 1 year, venous thromboembolism [VTE]-BLEED 30 days to 6 months, atrial fibrillation [AF]-BLEED 180 days, and Utah Bleeding Risk Score [UBRS] 3 years). Major bleeding was evaluated according to the International Society on Thrombosis and Haemostasis (ISTH), Interagency Registry for Mechanically Assisted Circulatory Support (INTERMACS), and INTERMACS + intracranial bleeding (INTERMACS+) criteria. The dotted line represents perfect calibration.

Figure 4 shows the calibration plots for the risk scores within their intended timeframes. Most risk scores demonstrated substantially lower predicted MB probabilities compared with the (estimated) observed probabilities. UBRS, according to all 3 MB criteria, and OBRI, based on INTERMACS criteria, exhibited significant underestimation for low predicted MB probabilities and slight overestimation for high predicted probabilities. Observed/expected ratios ranged from 1.38 to 36.48 (ISTH/INTERMACS+) and 1.18 to 31.56 (INTERMACS), intercepts from 0.49 to 4.38 (ISTH/INTERMACS+) and 0.21 to 4.04 (INTERMACS), and slopes from −0.03 to 0.31 (ISTH/INTERMACS+) and −0.11 to 0.50 (INTERMACS; Supplementary Table S7).

### 3.5 Sensitivity analyses

Most sensitivity analyses yielded comparable results with our main analyses.

#### 3.5.1 Cumulative incidence of MB among HeartWare patients as compared with HeartMate-3 patients

The maximum follow-up duration among patients with a HeartWare device (*n* = 94) was 4521 days as compared with 573 days among patients with HeartMate-3 (*n* = 10). Among the 2 devices, MB incidence was comparable according to ISTH and INTERMACS+ criteria. Although the incidence of MB was lower among HeartMate-3 patients per INTERMACS criteria, it was still high, and the difference between the devices was not statistically significant (Supplementary Table S8).

#### 3.5.2 Cumulative incidence of MB among patients prescribed ASA as compared with clopidogrel

The maximum follow-up duration among patients prescribed anticoagulation (VKA or heparin) combined with clopidogrel (*n* = 83) or ASA (*n* = 11) was 4521 and 784 days, respectively. While MB incidences were lower among patients prescribed ASA, a relevant proportion still experienced MB, and the difference between antiplatelet groups was not statistically significant (Supplementary Table S9).

#### 3.5.3 Complete case analysis

The complete case analysis included 94 patients. Baseline characteristics and cumulative MB incidence over time are depicted in Supplementary Table S10 and Figure S2. Findings demonstrated comparable discriminative capacity and calibration with the main analyses, as summarized in Supplementary Table S11 and Figure S3.

#### 3.5.4 Noncompeting risk analysis (mortality as a censoring event)

When considering mortality as a censoring event, predictive accuracies were consistent with those of the main analysis, as detailed in Supplementary Table S12 and Figure S4.

#### 3.5.5 Harrell *C*-index

Evaluation of the Harrell *C*-index confirmed similar discriminative performance to the main analysis, as summarized in Supplementary Table S13.

#### 3.5.6 MB beyond 14 days post-LVAD implantation with death and thrombolysis as competing events

The types of MB outside the 14 days post-LVAD implantation window and the cumulative incidence of MB and competing events (ie, death and thrombolytic therapy) over time are summarized in Supplementary Table S14 and Supplementary Figure S5. The predictive performance of the risk scores on their intended timeframe remained consistently poor, mirroring the results of the main analysis (Supplementary Table S15 and Figure S6).

#### 3.5.7 Correlation between risk scores and MB

**Figure 5:**
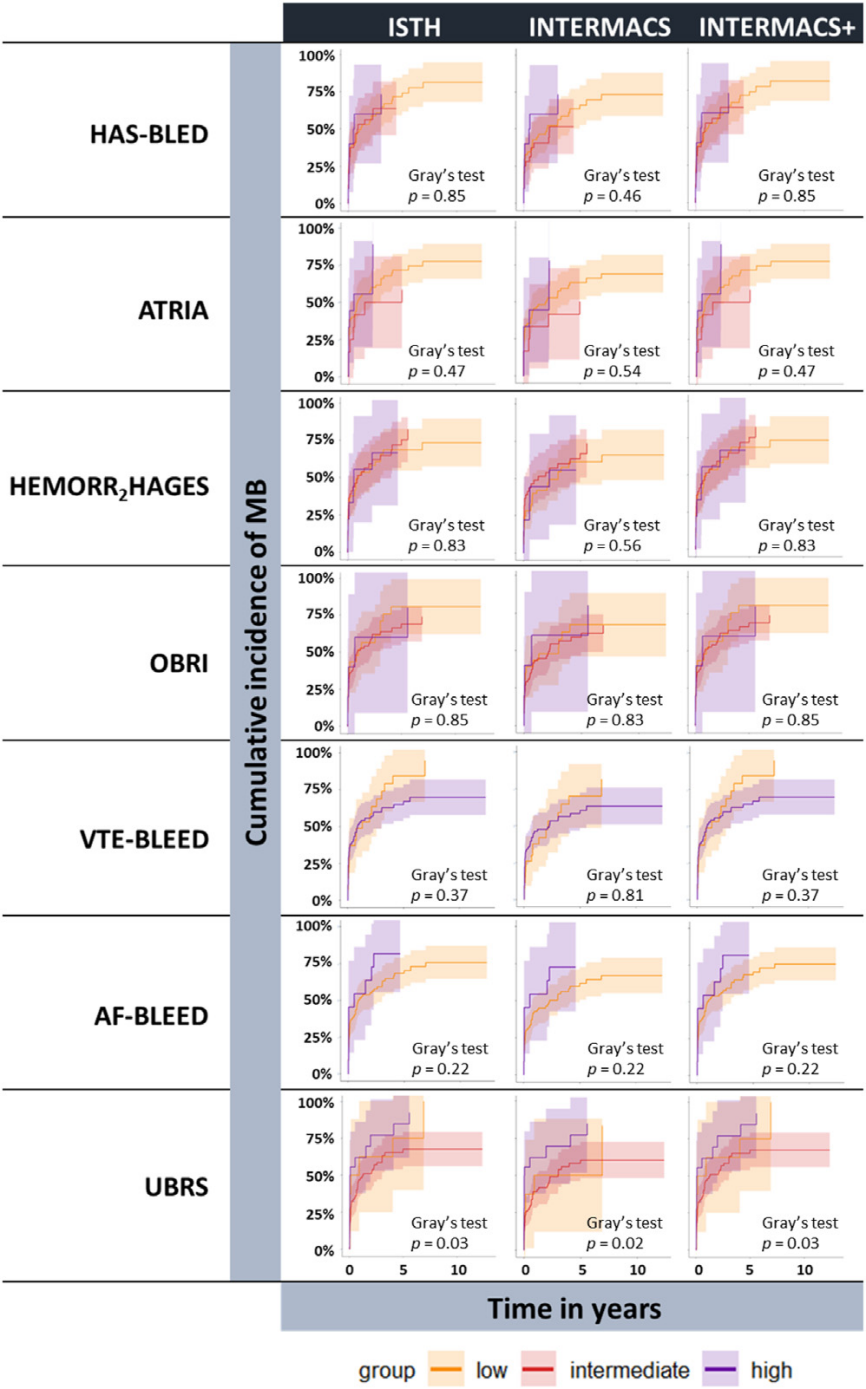
Cumulative incidence of major bleeding, with mortality as competing event, over time for risk groups (ie, low, intermediate, or high) as categorized by the risk scores. Major bleeding was evaluated according to the International Society on Thrombosis and Haemostasis (ISTH), Interagency Registry for Mechanically Assisted Circulatory Support (INTERMACS), and INTERMACS + intracranial bleeding (INTERMACS+) criteria. Group difference was assessed by Gray’s test (*P* < .05 for significance). AF-BLEED, atrial fibrillation-BLEED; ATRIA, Anticoagulation and Risk Factors in Atrial Fibrillation; HAS-BLED, Hypertension, Abnormal Renal/Liver Function, Stroke, Bleeding History or Predisposition, Labile INR, Elderly, Drugs/Alcohol Concomitantly; HEMORR_2_HAGES, Hepatic or Renal Disease, Ethanol Abuse, Malignancy, Older Age, Reduced Platelet Count or Function, Re-Bleeding, Hypertension, Anemia, Genetic Factors, Excessive Fall Risk and Stroke; OBRI, Outpatient Bleeding Risk Index; UBRS, Utah Bleeding Risk Score; VTE-BLEED, venous thromboembolism-BLEED.

There was no difference in cumulative MB incidences over time among risk score categories, except for UBRS (Gray’s test, *P* < .05; Figure 5). The Fine–Gray subdistribution hazard model revealed no association between any of the risk scores and MB incidences according to ISTH, INTERMACS, or INTERMACS+ criteria (Supplementary Table S16).

## 4 Discussion

We conducted a head-to-head comparative independent external validation study to evaluate the predictive performance of 7 bleeding risk scores in LVAD recipients, a clinically relevant high-risk target population for which these scores were not originally developed. All risk scores demonstrated poor predictive performance in predicting MB. HAS-BLED, HEMORR_2_HAGES, ATRIA, OBRI, VTE-BLEED, AF-BLEED, and UBRS had similar poor discriminative abilities, with AUC_t_ ranging between 0.48 (95% CI 0.40-0.56, ATRIA) and 0.56 (95% CI 0.47-0.65, UBRS). Reflective of the high risk of bleeding in this fragile population, all models were poorly calibrated, substantially underestimating MB events.

### 4.1 Current bleeding risk scores in LVAD recipients

Five previous studies explored the utility of bleeding risk scores in LVAD patients [[7], [8], [9],^11^,^26^]. Two lacked formal statistical evaluation of model performance, ie, discrimination and calibration assessments [7,8]. The remaining 3 studies assessed discrimination of 1 or more risk scores, yet none included calibration measurements [9,11,26].

#### 4.1.1 Conventional bleeding risk scores

Previous studies reported higher HAS-BLED and HEMORR_2_HAGES scores among patients who subsequently experienced a bleeding event [[7], [8], [9]]. However, we could not establish an association between risk scores and MB assessed by ISTH, INTERMACS, and INTERMACS+ criteria. Our findings regarding the predictive performance of conventional risk scores align with those of previous studies, reporting poor discriminative abilities (C-statistics ≤ 0.62) in LVAD recipients for HAS-BLED, HEMORR_2_HAGES, ATRIA, OBRI, and VTE-BLEED [9,26]. Additionally, our calibration results highlight extreme underprediction of MB risk in this population. This outcome was expected, considering that we validated the risk scores in a markedly different patient population compared with the cohorts from which the models were originally derived. The validated risk scores were developed in a population of patients with AF (eg, HAS-BLED, ATRIA, HEMORR_2_HAGES, and AF-BLEED), VTE-BLEED, or in all outpatients treated with warfarin (OBRI). The incidence rate of MB among patients with AF and VTE on VKA is approximately 2 per 100 PY, with a 5-year cumulative incidence of 6.3% [27]. However, LVAD patients face a vastly higher bleeding risk due to their anticoagulant regimen and post-LVAD hemostatic changes.

#### 4.1.2 Targeted bleeding risk score (UBRS)

The UBRS stands as a unique risk score, being the only score developed specifically for the LVAD population to date [10]. Nevertheless, our experience applying this score in our cohort was disappointing. Despite relatively higher C-statistics compared with the other validated risk scores, the predictive performance remained poor. Additionally, it is an interesting finding that even the UBRS underestimated the MB risk for most patients in our cohort, a finding that is thus less likely explained by case mix heterogeneity.

Importantly, the UBRS was originally designed to predict GIB rather than MB. When models are validated for outcomes other than those they were originally intended for, suboptimal performance cannot be solely attributed to the model’s limitations. Additionally, 97% of LVADs in our cohort were implanted as destination therapy compared with 34% in the UBRS development cohort. The predominance of patients ineligible for cardiac transplantation implies a population with poorer health status, potentially leading to a heightened bleeding susceptibility. These factors may have contributed to the poor predictive performance of URBS in our cohort, affecting both its discriminative ability and its calibration. Nevertheless, in previous external validation studies focusing on the ability to predict 3-year GIB, unsatisfactory discrimination was reported as well (C-statistics ≤ 0.59) [11,26].

### 4.2 Implications

Despite improved thromboembolic outcomes with the newer generation HeartMate-3 device, MB complications remain a major concern regardless of the LVAD type implanted [5]. A multicenter propensity-score matching study, including 588 HeartWare and 433 HeartMate-3 recipients, reported a 40% to 50% cumulative 2-year MB incidence in both groups (*P* = .49) [6]. To mitigate MB risk, previous studies explored the safety of minimizing anticoagulant therapy in selected patients. The Minimal AnticoaGulation EvaluatioNTo aUgment heMocompatibility (MAGENTUM 1) pilot trial (*n* = 15, HeartMate-3) suggested that a low-intensity anticoagulation regimen (warfarin, target INR 1.5-1.9, with ASA) is safe with regard to thromboembolic outcomes within the first 6 months post-LVAD implantation [28]. Two observational studies (*n* = 161 and *n* = 60, HeartMate-3) reported that reduced antithrombotic therapy (VKA-only, ASA-only, or no antithrombotic therapy at all) may reduce bleeding complications without increasing thromboembolic risk compared with standard anticoagulant care [29,30]. However, the prospective observational United States STudy of Reduced Anti-Coagulation/Anti-platelEt Therapy in Patients with the HeartMate II Left Ventricular Assist System (US-TRACE) (*n* = 100, HeartMate-2) concluded that discontinuing ASA, VKA, or both may increase thromboembolic complications (incidence pump thrombosis 7%, ischemic stroke 6%) with persistent bleeding events (approximately 40% among the 3 treatment groups) at 1-year follow-up [31]. Of note, the US-TRACE did not make direct comparisons with standard anticoagulant care, and thromboembolic events mainly occurred in patients treated with ASA only or without any antithrombotic therapy. The Antiplatelet Removal and Hemocompatibility Events With the HeartMate 3 Pump (ARIES-HM3) trial (*n* = 628) comparing HeartMate-3 recipients randomized to receive either placebo or ASA alongside VKA therapy (target range, 2.0-3.0) demonstrated that VKA-only therapy is noninferior to an ASA-containing regimen and is associated with reduced bleeding events without an increase in thromboembolic events [32]. Overall, it is reasonable to hypothesize that patients at high risk of MB may benefit from upfront reduced antithrombotic treatment strategies. However, patient-tailored anticoagulant care requires an accurate risk assessment tool for identifying those at highest MB risk.

Unfortunately, the results of the present study indicate that current risk scores are not useful in predicting MB in LVAD patients. Common predictors in current risk models include higher age, hypertension, (chronic) kidney disease, history of stroke, prior bleeding, and anemia. Interestingly, a recent systematic review and meta-analysis found no significant associations between GIB and common predictors (eg, age, sex, hypertension, chronic kidney disease, and diabetes) in LVAD recipients [33]. This observation, together with the remarkable differences in predictor distribution within the derivation cohorts as compared with the present LVAD cohort, lead us to question which factors genuinely predict MB in patients with an LVAD. Hemostatic changes post-LVAD implantation, particularly acquired von Willebrand disease, subsequent angiodysplasia, and platelet dysfunction, are believed to increase the bleeding risk [3,34]. Exploring biochemical measurements as predictive indicators for MB in future studies would be valuable.

### 4.3 Strengths and limitations

Our study was characterized by several methodological strengths. Our main strength lies in validating all risk scores on the same clinically relevant outcome, facilitating a head-to-head model performance comparison. Additionally, we evaluated MB outcomes according to widely accepted criteria (ISTH and INTERMACS) and extended the INTERMACS criteria with intracranial hemorrhages (INTERMACS+ criteria). Secondly, to ensure the models were validated as originally intended, we aligned our predictor definitions with those specified in the development studies and validated the scores within their intended timeframes. Additionally, all patients had complete follow-up data, except for one who was transferred to another hospital, ensuring data reliability. Furthermore, unlike prior studies, we evaluated both discrimination and calibration, providing a comprehensive understanding of the predictive performance. The accuracy of risk estimates, alongside discriminative ability, is crucial in clinical decision making, as calibration assesses whether predicted probabilities align with observed outcomes. Lastly, given the relatively high mortality rates among LVAD recipients, considering mortality prior to MB as a competing event allowed us to appropriately estimate the cumulative incidence of MB by acknowledging that deceased patients could not subsequently experience an MB.

Several limitations should be considered in interpreting our results. The main constraint was the relatively small sample size, combined with a high incidence of MB events. The skewed ratio of events vs nonevents (ie, patients developing an MB vs those experiencing neither MB nor a competing event) might have had an impact on the validity and accuracy of the predictive performance measurements. Second, data on INR and genetic variants of CYP2C9 were not available. A preimplantation labile INR might be a valuable predictor of MB, which warrants caution in interpreting the predictive accuracy of the HAS-BLED risk score. Genetic variants of CYP2C9, on the other side, were unavailable in the HEMORR_2_HAGES development cohort as well, so the absence of this data should not significantly impact the validity of our results. Furthermore, a small proportion of patients had missing data on MB history (*n* = 5; 4.8%) and/or MPAP (*n* = 5; 4.8%). In these cases, a score of zero was assigned to the variable. Although this might have potentially affected the predictive performance measures, it mirrors daily clinical practice when calculating a risk score. Additionally, complete case analyses yielded similar results. Third, the majority of patients in our cohort received a HeartWare device (90.4%), which was withdrawn from the market in 2021. However, we anticipate that our findings would extrapolate to patients with the newer generation HeartMate device. The current guideline of the International Society for Heart and Lung Transplantation (2023) still recommends treatment with VKA targeting an INR of 2.0 to 3.0 combined with ASA for both HeartWare and HeartMate-3 [2]. Only 8 cases of MB in our study were directly related to intensified anticoagulant therapy or systemic thrombolysis following pump thrombosis, underscoring the continued relevance of bleeding risk prediction in patients with less thrombogenic LVADs. Of note, most patients (70%) received clopidogrel in addition to VKA, which is regarded as a more potent antiplatelet agent than ASA. Additionally, most patients (97%) were ineligible for cardiac transplantation and were implanted with an LVAD as destination therapy. Unfortunately, conducting sensitivity analyses within HeartMate-3 patients, patients treated with ASA instead of clopidogrel, or patients with a device strategy other than destination therapy was not feasible due to the limited number of patients in these groups. Nonetheless, cumulative MB incidences among HeartMate-3 patients and patients treated with ASA alongside anticoagulation were still high and comparable to patients with a HeartWare device or patients treated with anticoagulation combined with clopidogrel, respectively. Lastly, the validated risk scores lack a formula for calculating predicted probabilities. Instead, we used bleeding incidences or rates reported for each score sum in the original articles as proxies for predicted probabilities. This limitation is not unique to our study and is a common constraint in validation studies. Nevertheless, it reflects how these risk scores are used in medical practice.

## 5 Conclusion

Our study underscores a significant limitation in the current validated bleeding risk scores when applied to LVAD recipients. These scores, in their existing forms, fail to predict MB events in this high-risk population and should therefore not be used. This observation highlights the need for a more accurate risk assessment tool to reliably identify LVAD patients at high MB risk. Such a tool could guide patient-tailored antithrombotic therapy, mitigating MB risk and improving overall patient care. Further research on this topic is crucial to address this gap in knowledge in clinical practice.
