# Erratum to ‘Performance of Risk Scores in Predicting Major Bleeding in Left Ventricular Assist Device (LVAD) Recipients: a Comparative External Validation. ’[Res Pract Thromb Haemost. 2024;8:e102437]

**DOI:** 10.1016/j.rpth.2024.102558

**Published:** 2024-08-30

**Authors:** S.F.B. van der Horst, Y. de Jong, N. van Rein, J.W. Jukema, M. Palmen, E. Janssen, E.F. Bonneville, F.A. Klok, M.V. Huisman, L.F. Tops, P.L. den Exter

**Affiliations:** 1 Department of Medicine—Thrombosis and Hemostasis, Leiden University Medical Center, Leiden, The Netherlands; 2 Department of Clinical Epidemiology, Leiden University Medical Center, Leiden, The Netherlands; 3 Department of Clinical Pharmacy and Toxicology, Leiden University Medical Center, Leiden, The Netherlands; 4 Department of Cardiology, Leiden University Medical Center, Leiden, The Netherlands; 5 Department of Thoracic Surgery, Leiden University Medical Center, Leiden, The Netherlands; 6 Department of Biomedical Data Sciences, Leiden University Medical Center, Leiden, The Netherlands

The publisher regrets having introduced errors in the abstract of this article, during the production process. Specifically, the abbreviations/acronyms for the validated risk scores were incorrectly presented. The following corrections should be noted:

1. "venous thromboembolism score" should be "venous thromboembolism (VTE)-BLEED score."
2. "atrial fibrillation score" should be "atrial fibrillation (AF)-BLEED score."
3. The other scores should include their respective abbreviations/acronyms in parentheses in the Methods section of the abstract, which should have been used consistently in the Results section of the abstract: Hypertension, Abnormal Renal/Liver Function, Stroke, Bleeding History or Predisposition, Labile INR, Elderly, Drugs/Alcohol Concomitantly (HAS-BLED) score; Hepatic or Renal Disease, Ethanol Abuse, Malignancy, Older Age, Reduced Platelet Count or Function, Re-Bleeding, Hypertension, Anemia, Genetic Factors, Excessive Fall Risk and Stroke (HEMORR2HAGES) score; Anticoagulation and Risk Factors in Atrial Fibrillation (ATRIA) score; Outpatient Bleeding Risk Index (OBRI); and Utah Bleeding Risk Score (UBRS).

The publisher would like to apologise for any inconvenience caused.

