# Supplementary material for: Performance of risk scores in predicting major bleeding in left ventricular assist device recipients: a comparative external validation
Source: Res Pract Thromb Haemost. 2024 May 6;8(4):102437. doi: 10.1016/j.rpth.2024.102437 (PMC11215111; doi:10.1016/j.rpth.2024.102437)
Supplement: Supplementary Tables S1-S16 and Figures S1-S6 [file mmc1.docx]

**Supplementary Material for:**

**Performance of Risk Scores in Predicting Major Bleeding in Left Ventricular Assist Device (LVAD) Recipients: a Comparative External Validation**

S.F.B. van der Horst^1^, Y. de Jong^1,2^, N. van Rein^2,3^, J.W. Jukema^4^, M. Palmen^5^, E. Janssen^4^, E.F. Bonneville^6^, F.A. Klok^1^, M.V. Huisman^1^, L.F. Tops^4^, P.L. den Exter^1^

^1^ Department of Medicine - Thrombosis and Hemostasis, Leiden University Medical Center, Leiden, The Netherlands

^2^ Department of Clinical Epidemiology, Leiden University Medical Center, Leiden, Netherlands

^3^ Department of Clinical Pharmacy and Toxicology, Leiden University Medical Center, Leiden, Netherlands

^4^ Department of Cardiology, Leiden University Medical Center, Leiden, Netherlands

^5^ Department of Thoracic surgery, Leiden University Medical Center, Leiden, Netherlands
^6^ Department of Biomedical Data Sciences, Leiden University Medical Center, Leiden, the Netherlands

**INDEX**

CHECKLISTS

Page 3-4 **STROBE Statement**: Checklist of items that should be included in reports of cohort studies

Page 5 **TRIPOD Checklist**: Prediction Model Development and Validation

RISK SCORE CHARACTERISTICS

Page 6 **Table S1** Outcome definitions according to ISTH, INTERMACS and INTERMACS+

Page 7 **Table S2** Included risk scores - characteristics of derivation cohorts

Page 8-11 **Table S3** Predictor definitions risk scores

Page 12 **Table S4** Predicted probabilities for each risk score

Page 12 Strategy for converting event rates (EVR) to approximated cumulative

incidences (CI)

SUPPLEMENTARY DATA

Page 13 **Table S5** Cumulative incidences and incidence rates of major bleeding

Page 14 **Figure S1** Distribution of scores and risk-categories

Page 15-16 **Table S6** Distribution of predictors and comparison with derivation cohorts

Page 17 **Table S7** Calibration measurements of each risk score on their intended timeframe, competing risk analysis

Page 18 **Table S8** Cumulative incidence of MB among HeartWare as compared to HeartMate-3 patients

Page 19 **Table S9** Cumulative incidences of MB within patients initially prescribed clopidogrel vs ASA

Page 20 **Table S10** Clinical characteristics of the entire cohort vs the complete case cohort

Page 21 **Figure S2** Cumulative incidence plot of major bleeding and mortality (competing event), complete-case analysis

Page 22 **Table S11** Discriminative ability of each risk score on their intended timeframe, complete-case analysis

Page 22 **Figure S3** Calibration plots of each risk score on their intended timeframe, complete-case analysis

Page 23 **Table S12** Discriminative ability of each risk score on their intended timeframe, non-competing risk analysis

Page 23 **Figure S4** Calibration plots of each risk score on their intended timeframe, non-competing risk analysis

Page 24 **Table S13** Harrell C-index for each risk score on their own timeframe

Page 25 **Table S14** Types of major bleeding beyond 14 days post-LVAD implantation

Page 25 **Figure S5** Cumulative incidence plot of major bleeding beyond 14 days post-LVAD implantation, with mortality and thrombolysis as competing events

Page 26 **Table S15** Discriminative ability of each risk score on their intended timeframe, only including major bleeding beyond 14 days post-LVAD implantation and with thrombolysis and death as competing events

Page 26 **Figure S6** Calibration plots of each risk score on their intended timeframe, only including major bleeding beyond 14 days post-LVAD implantation and with thrombolysis and death as competing events

Page 27 **Table S11** Univariate Fine-Gray Subdistribution Hazards Model (entire follow-up duration)

Page 28 **References**

**STROBE Statement—Checklist of items that should be included in reports of *cohort studies***

|  | **Item No** | **Recommendation** | **Page No** |
| --- | --- | --- | --- |
| **Title and abstract** | 1 | (*a*) Indicate the study’s design with a commonly used term in the title or the abstract | 1 |
|  |  | (*b*) Provide in the abstract an informative and balanced summary of what was done and what was found | 2 |
| **Introduction** | | | |
| Background/rationale | 2 | Explain the scientific background and rationale for the investigation being reported | 3 |
| Objectives | 3 | State specific objectives, including any prespecified hypotheses | 3-4 |
| **Methods** | | | |
| Study design | 4 | Present key elements of study design early in the paper | 4 |
| Setting | 5 | Describe the setting, locations, and relevant dates, including periods of recruitment, exposure, follow-up, and data collection | 4 |
| Participants | 6 | (*a*) Give the eligibility criteria, and the sources and methods of selection of participants. Describe methods of follow-up | 5 |
|  |  | (*b*) For matched studies, give matching criteria and number of exposed and unexposed |  |
| Variables | 7 | Clearly define all outcomes, exposures, predictors, potential confounders, and effect modifiers. Give diagnostic criteria, if applicable | 5 - 6 |
| Data sources/ measurement | 8* | For each variable of interest, give sources of data and details of methods of assessment (measurement). Describe comparability of assessment methods if there is more than one group | 5 - 6 |
| Bias | 9 | Describe any efforts to address potential sources of bias | 5 - 6 |
| Study size | 10 | Explain how the study size was arrived at | 4 |
| Quantitative variables | 11 | Explain how quantitative variables were handled in the analyses. If applicable, describe which groupings were chosen and why | 6 - 8 |
| Statistical methods | 12 | (*a*) Describe all statistical methods, including those used to control for confounding | 6 – 8 |
|  |  | (*b*) Describe any methods used to examine subgroups and interactions | 6 – 8 |
|  |  | (*c*) Explain how missing data were addressed | 6 – 7 |
|  |  | (*d*) If applicable, explain how loss to follow-up was addressed | 4, 7 |
|  |  | (*e*) Describe any sensitivity analyses | 7 - 8 |
| **Results** | | |  |
| Participants | 13* | (a) Report numbers of individuals at each stage of study—eg numbers potentially eligible, examined for eligibility, confirmed eligible, included in the study, completing follow-up, and analysed | 8 – 9 |
|  |  | (b) Give reasons for non-participation at each stage | N.A. |
|  |  | (c) Consider use of a flow diagram | N.A. |
| Descriptive data | 14* | (a) Give characteristics of study participants (eg demographic, clinical, social) and information on exposures and potential confounders | 8 – 10 |
|  |  | (b) Indicate number of participants with missing data for each variable of interest | 6, 11 |
|  |  | (c) Summarise follow-up time (eg, average and total amount) | 9 |
| Outcome data | 15* | Report numbers of outcome events or summary measures over time | 9 - 10 |
| Main results | 16 | (*a*) Give unadjusted estimates and, if applicable, confounder-adjusted estimates and their precision (eg, 95% confidence interval). Make clear which confounders were adjusted for and why they were included | 9 - 11 |
|  |  | (*b*) Report category boundaries when continuous variables were categorized | 10 |
|  |  | (*c*) If relevant, consider translating estimates of relative risk into absolute risk for a meaningful time period | N.A. |
| Other analyses | 17 | Report other analyses done—eg analyses of subgroups and interactions, and sensitivity analyses | 11 - 12 |
| **Discussion** | | | |
| Key results | 18 | Summarise key results with reference to study objectives | 12 |
| Limitations | 19 | Discuss limitations of the study, taking into account sources of potential bias or imprecision. Discuss both direction and magnitude of any potential bias | 16 - 17 |
| Interpretation | 20 | Give a cautious overall interpretation of results considering objectives, limitations, multiplicity of analyses, results from similar studies, and other relevant evidence | 12 - 15 |
| Generalisability | 21 | Discuss the generalisability (external validity) of the study results | 15 - 17 |
| **Other information** | | | |
| Funding | 22 | Give the source of funding and the role of the funders for the present study and, if applicable, for the original study on which the present article is based | 17 |

**TRIPOD Checklist: Prediction Model Development and Validation**

| Section/Topic | Item |  | Checklist Item | Page |
| --- | --- | --- | --- | --- |
| Title and abstract | | | | |
| Title | 1 | D;V | Identify the study as developing and/or validating a multivariable prediction model, the target population, and the outcome to be predicted. | 1 |
| Abstract | 2 | D;V | Provide a summary of objectives, study design, setting, participants, sample size, predictors, outcome, statistical analysis, results, and conclusions. | 2 |
| Introduction | | | | |
| Background and objectives | 3a | D;V | Explain the medical context (including whether diagnostic or prognostic) and rationale for developing or validating the multivariable prediction model, including references to existing models. | 3 |
|  | 3b | D;V | Specify the objectives, including whether the study describes the development or validation of the model or both. | 4 |
| Methods | | | | |
| Source of data | 4a | D;V | Describe the study design or source of data (e.g., randomized trial, cohort, or registry data), separately for the development and validation data sets, if applicable. | 4 |
|  | 4b | D;V | Specify the key study dates, including start of accrual; end of accrual; and, if applicable, end of follow-up. | 4 |
| Participants | 5a | D;V | Specify key elements of the study setting (e.g., primary care, secondary care, general population) including number and location of centres. | 4 |
|  | 5b | D;V | Describe eligibility criteria for participants. | 4 |
|  | 5c | D;V | Give details of treatments received, if relevant. | N.A. |
| Outcome | 6a | D;V | Clearly define the outcome that is predicted by the prediction model, including how and when assessed. | 5 |
|  | 6b | D;V | Report any actions to blind assessment of the outcome to be predicted. | 6 |
| Predictors | 7a | D;V | Clearly define all predictors used in developing or validating the multivariable prediction model, including how and when they were measured. | 5, 6 |
|  | 7b | D;V | Report any actions to blind assessment of predictors for the outcome and other predictors. | 6 |
| Sample size | 8 | D;V | Explain how the study size was arrived at. | 4 |
| Missing data | 9 | D;V | Describe how missing data were handled (e.g., complete-case analysis, single imputation, multiple imputation) with details of any imputation method. | 6 |
| Statistical analysis methods | 10a | D | Describe how predictors were handled in the analyses. | N.A. |
|  | 10b | D | Specify type of model, all model-building procedures (including any predictor selection), and method for internal validation. | N.A. |
|  | 10c | V | For validation, describe how the predictions were calculated. | 6 |
|  | 10d | D;V | Specify all measures used to assess model performance and, if relevant, to compare multiple models. | 7, 8 |
|  | 10e | V | Describe any model updating (e.g., recalibration) arising from the validation, if done. | N.A. |
| Risk groups | 11 | D;V | Provide details on how risk groups were created, if done. | 6 |
| Development vs. validation | 12 | V | For validation, identify any differences from the development data in setting, eligibility criteria, outcome, and predictors. | 6, 10 |
| Results | | | | |
| Participants | 13a | D;V | Describe the flow of participants through the study, including the number of participants with and without the outcome and, if applicable, a summary of the follow-up time. A diagram may be helpful. | 9 - 11 |
|  | 13b | D;V | Describe the characteristics of the participants (basic demographics, clinical features, available predictors), including the number of participants with missing data for predictors and outcome. | 6, 9 |
|  | 13c | V | For validation, show a comparison with the development data of the distribution of important variables (demographics, predictors and outcome). | 10 |
| Model development | 14a | D | Specify the number of participants and outcome events in each analysis. | N.A. |
|  | 14b | D | If done, report the unadjusted association between each candidate predictor and outcome. | N.A. |
| Model specification | 15a | D | Present the full prediction model to allow predictions for individuals (i.e., all regression coefficients, and model intercept or baseline survival at a given time point). | N.A. |
|  | 15b | D | Explain how to the use the prediction model. | N.A. |
| Model performance | 16 | D;V | Report performance measures (with CIs) for the prediction model. | 10-12 |
| Model-updating | 17 | V | If done, report the results from any model updating (i.e., model specification, model performance). | N.A. |
| Discussion | | | | |
| Limitations | 18 | D;V | Discuss any limitations of the study (such as nonrepresentative sample, few events per predictor, missing data). | 16, 17 |
| Interpretation | 19a | V | For validation, discuss the results with reference to performance in the development data, and any other validation data. | 13, 14 |
|  | 19b | D;V | Give an overall interpretation of the results, considering objectives, limitations, results from similar studies, and other relevant evidence. | 13 - 17 |
| Implications | 20 | D;V | Discuss the potential clinical use of the model and implications for future research. | 15, 16 |
| Other information | | | | |
| Supplementary information | 21 | D;V | Provide information about the availability of supplementary resources, such as study protocol, Web calculator, and data sets. | Online form |
| Funding | 22 | D;V | Give the source of funding and the role of the funders for the present study. | 17 |

*Items relevant only to the development of a prediction model are denoted by D, items relating solely to a validation of a prediction model are denoted by V, and items relating to both are denoted D;V. We recommend using the TRIPOD Checklist in conjunction with the TRIPOD Explanation and Elaboration document.

| **Supplementary Table S1. Outcome definitions according to ISTH, INTERMACS and INTERMACS+ criteria** | | |
| --- | --- | --- |
| ISTH[1] | INTERMACS[2] | INTERMACS+[2] |
| Symptomatic presentation and:   1. Fatal bleeding, **AND/OR** 2. Bleeding in a critical area or organ, such as intracranial, intraspinal, intraocular, retroperitoneal, intra-articular or pericardial, or intramuscular with compartment syndrome, **AND/OR** 3. Bleeding causing a fall in hemoglobin level of 20 g L^−1^ (1.24 mmol L^−1^) or more, or leading to transfusion of two or more units of whole blood or red cells. | **MCS-ARC bleeding type 3, 4 or 5:**  Type 3:   - Type 3a: Overt bleeding accompanied by hemoglobin drop of 3 to < 5 g/dl or (1.86−3.1 mmol/liter SI units) (provided hemoglobin drop is related to bleed) **AND/OR** any transfusion with overt bleeding - Type 3b: Overt bleeding plus hemoglobin drop 5 g/dl ((3.1 mmol/liter) or greater (provided hemoglobin drop is related to bleed) **AND/OR** cardiac tamponade **AND/OR** bleeding requiring surgical intervention for control (excluding dental/nasal/skin/hemorrhoid) **AND/OR** bleeding requiring intravenous vasoactive agents   Type 4: VAD implantation-related bleeding (includes concomitant cardiac or non-cardiac surgical procedures)   - Reoperation after the closure of incision or incisions used to implant the VAD to control bleeding - ≥ 50 kg: ≥ 4U PRBC within any 48 hours during the first 7 days post-implant. - < 50 kg: ≥ 20 cm3/kg PRBC within any 24 hours during the first 7 days post-implant. - Chest tube output > 2 liters within 24 hours.   Type 5: Fatal bleeding   - Type 5a: Probable fatal bleeding; no autopsy or imaging confirmation but clinically suspicious - Type 5b: Definite fatal bleeding; overt bleeding or autopsy or imaging confirmation | **MCS-ARC bleeding type 3, 4 or 5**  **AND/OR**  **Intracranial hemorrhage**: (acute) neurological symptoms with documented intracranial bleeding on imaging or post-mortem assessment |

| **Supplementary Table S2. Included risk scores - characteristics of derivation cohorts** | | | | |
| --- | --- | --- | --- | --- |
| **Study** | **Name risk score** | **Population** | **Outcome** | **Timeframe** |
| **Pisters et al. 2010**[3] | HAS-BLED | Adults with AF  N: 3456  Age, mean (SD): 66.8±12.8  Sex, women: 41% | Major bleeding: any bleeding, with the exception of hemorrhagic stroke:  - Requiring hospitalization, and/or - Causing a decrease in hemoglobin level of >2 g/L, and/or - Requiring a blood transfusion  Number of events (%): 53 (1.5) | Bleeds per 100PY at 1-year |
| **Gage et al. 2006**[4] | HEMORR_2_HAGES | Elderly patients with AF  N: 3791  Age, mean: 80.2  Sex, women: 57%^a^ | Time to hospitalization for bleeding (major bleeding based on ICD-9-CM codes)  Number of events (%): 162 (4.3) | Bleeds per 100PY at a maximum follow-up of 1000 days |
| **Fang et al. 2011**[5] | ATRIA | Adults with AF  N: 6123  Age ≥75 years: 53.5%^a^  Sex, women: 41.8%^a^ | Major hemorrhage: fatal, requiring transfusion of ≥2 units packed blood cells, or hemorrhage into a critical anatomic site (e.g., intracranial, retroperitoneal)  Number of events (%): 307 (5.0) | Bleeds per 100PY, annualized hemorrhage rate |
| **Beyth et al. 1998**[6] | Outpatient Bleeding Risk Index (OBRI) | Outpatients treated with warfarin  N: 556  Age, mean (SD): 61 ± 14  Sex, women: 53% | Major bleeding: overt bleeding that led to the loss of at least 2.0 units in 7 days or less, or was otherwise life-threatening (eg, intracranial bleeding)  Number of events: 65 (11.7) | Cumulative bleeding incidence at 12 months |
| **Klok et al. 2016**[7] | VTE-BLEED | Patients with VTE using dabigatran  N: 2553  Age ≥60 years: 41%  Sex, women: 40.5%^a^ | Major bleeding (ISTH criteria)  Number of events (%): 37 (1.4) | Absolute bleeding incidence between 30 days and 6 months after start of anticoagulant therapy |
| **Chu et al. 2021**[8] | AF-BLEED | Patients with AF  N: 18,040  Age, mean (SD): 71.4±8.6  Sex, women: 36.4% | Major bleeding (ISTH criteria)  Number of events (%): 1014 (5.6) | Absolute bleeding incidence during first 180 days |
| **Yin et al. 2018**[9] | Utah Bleeding Risk Score (UBRS) | Patients with an LVAD  N: 351  Age, median (IQ1 – IQ3): 59 (48 – 67)  Sex, women: 18% | Gastrointestinal bleeding (clinical evidence for bleeding, resulting in:  - Hb decrease ≥2 g/dL AND  - death, or surgical intervention, or hospitalization or transfusion of packed red blood cells)  Number of events (%): 120 (34.2) | Cumulative incidence of bleeding at 3 years |
| Abbreviations: N: number, SD: standard deviation, PY: patient-years, AF: atrial fibrillation | | | | |

| **Supplementary Table S3. Predictor definitions risk scores** | | | | |
| --- | --- | --- | --- | --- |
| **Risk score** | **Predictor definitions** | | | **Point** |
|  | Development cohort | | LUMC-LVAD cohort |  |
| **HAS-BLED score**[3] | | | | |
| Hypertension | Uncontrolled blood pressure, systolic >160 mmHg | | Systolic blood pressure >160 mmHg prior LVAD implantation | 1 |
| Renal disease | - Chronic dialysis, or  - History of renal transplant, or  - Serum creatinine ≥200µmol/L / ≥2.26 mg/dL | | - Dialysis, or  - History of renal transplant, or  - Serum creatinine ≥200µmol/L / ≥2.26 mg/dL | 1 |
| Liver disease | - Chronic hepatic disease (e.g., cirrhosis), or  - Bilirubin >2x ULN and AST/ALT/AP >3x ULN | | - Cirrhosis, or  - Bilirubin >2x ULN (34 µmol/L / 1.99 mg/dL) and AST/ALT >3x ULN (AST > 105 U/L, ALT > 135 U/L) | 1 |
| Stroke history | Not defined | | History of ischemic stroke | 1 |
| Prior major bleeding or predisposition to bleeding | - Prior major bleeding (not defined), or  - Predisposition to bleeding (= anemia, not defined) | | - History of major bleeding (according to ISTH criteria), or  - Anemia: hemoglobin < 12 g/dL / 7.45 mmol/L in men or <11 g/dL / 6.83 mmol/L in women | 1 |
| Labile INR | Time in therapeutic range <60% | | N.A. | 1 |
| Age | > 65 years | | > 65 years | 1 |
| Medication usage predisposing to bleeding | Aspirin, clopidogrel or NSAIDs | | Use of antiplatelet drugs prior to LVAD implantation | 1 |
| Alcohol use | ≥8 alcoholic drinks/week | | ≥8 alcoholic drinks/week | 1 |
| **HEMORR_2_HAGES score**[4] | | |  |  |
| Hepatic or renal disease | | - Hepatic disease: end-stage liver disease or cirrhosis, or  - Renal disease: some QIOs included only end-stage renal disease; others included patients with a creatinine >2.5 mg/dL / >221.05 µmol/L | - Cirrhosis, or  - Dialysis, or  - Serum creatinine >2.5 mg/dL / >221.05 µmol/L | 1 |
| Alcohol abuse | | ICD-9-CM codes 291.0-2, 303.x, 305.0x, 571.0-3, 535.3 | Modest alcohol use: ≥2 alcoholic drinks / day | 1 |
| Malignancy history | | ICD-9-CM codes: 141-172, 174-208 | Documented history of cancer or active cancer | 1 |
| Older age | | > 75 years | > 75 years | 1 |
| Reduced platelet count or function | | - Aspirin use  - Thrombocytopenia (not defined)  - QIO review captured blood dyscrasias (e.g. hemophilia) in some states | - Use of antiplatelet drugs prior to LVAD implantation, or  - Thrombocytopenia (thrombocyte count <150 x 10^9^/L), or  - Documented dyscrasia (e.g. hemophilia) | 1 |
| Rebleeding risk | | Prior bleeding (not defined) | Any prior documented bleeding event | 2 |
| Hypertension | | Uncontrolled, ICD-9-CM codes: 401.0, 402.0x, 403.0x, 404.0x, 405.0x | Malignant hypertension prior LVAD implantation (systolic blood pressure > 180 or diastolic blood pressure >120 mmHg) | 1 |
| Anemia | | ICD-9-CM codes: 280.x, 281.x, 282.0-4, 282.60, 282.69, 283.x, 284.x, 285.x | Hemoglobin < 12 g/dL / 7.45 mmol/L in men or <11 g/dL / 6.83 mmol/L in women | 1 |
| Genetic factors | | CYP 2C9 single-nucleotide polymorphisms, N.A. in development cohort | N.A. | 1 |
| Excessive fall risk | | High risk of falling, dementia, Parkinson disease, or psychiatric diseases | Documented history of dementia, Parkinson disease or psychiatric diseases | 1 |
| Stroke history | | Chart review or ICD-9-CM codes 434-436 in the primary position | History of ischemic stroke | 1 |
| **ATRIA score**[5] | | | | |
| Anemia | | Hb <12 g/dL in men or <11 g/dL in women | Hemoglobin < 12 g/dL / 7.45 mmol/L in men or <11 g/dL / 6.83 mmol/L in women | 3 |
| Severe renal disease | | Glomerular filtration rate <30 mL/min or dialysis-dependent | Dialysis, or eGFR <30 mL/min | 3 |
| Age | | ≥75 years | ≥75 years | 2 |
| History of bleeding | | Any prior hemorrhage diagnosis | Any prior documented bleeding event | 1 |
| Hypertension | | Diagnosed hypertension (not defined) | Documented history of hypertension | 1 |
| **Outpatient Bleeding Risk (OBRI) score**[6] | | | | |
| Age | | ≥65 years | ≥65 years | 1 |
| History of gastro-intestinal bleeding | | Not defined | Any documented history of gastro-intestinal bleeding | 1 |
| Stroke history | | Not defined | History of ischemic stroke | 1 |
| Comorbidities | | Recent myocardial infarction (timeframe not defined), creatinine >1.5 mg/dL / >132.6 µmol/L, diabetes or hematocrit <30% | Myocardial infarction < 3 months prior LVAD implantation, serum creatinine >1.5 mg/dL / >132.6 µmol/L, diabetes or hematocrit <30% | 1 |
| **VTE-BLEED score**[7] | | | | |
| Active cancer | | Not defined | Confirmed diagnosis of cancer (by histology or adequate imaging modality), other than basal-cell or squamous-cell carcinoma of the skin alone with one of the following:  - locally active, regionally invasive, or metastatic cancer at the time of LVAD implantation, and/or  - Currently receiving or having received anticancer therapy  (radiotherapy, chemotherapy, hormonal therapy, any kind of  targeted therapy or any other anticancer therapy) in the last 6  months | 2 |
| Male patient with uncontrolled hypertension | | Male patient with blood pressure ≥140 mmHg systolic | Male patient with blood pressure ≥140 mmHg systolic | 1 |
| Anemia | | Hemoglobin <12 g/dL in men or <11 g/dL in women | Hemoglobin < 12 g/dL / 7.45 mmol/L in men or <11 g/dL / 6.83 mmol/L in women | 1.5 |
| History of bleeding | | Prior major or non-major clinically relevant bleeding event, rectal  bleeding, frequent nose bleeding or hematuria | Documented history of major or non-major clinically relevant bleeding event, rectal  bleeding, frequent nose bleeding or hematuria | 1.5 |
| Renal dysfunction | | eGFR 30-60 ml/min | eGFR 30-60 ml/min | 1.5 |
| Age | | ≥60 years | ≥60 years | 1.5 |
| **AF-BLEED score**[8] | | | | |
| Active cancer | | Not defined | Confirmed diagnosis of cancer (by histology or adequate imaging modality), other than basal-cell or squamous-cell carcinoma of the skin alone with one of the following:  - locally active, regionally invasive, or metastatic cancer at the time of LVAD implantation, and/or  - Currently receiving or having received anticancer therapy  (radiotherapy, chemotherapy, hormonal therapy, any kind of  targeted therapy or any other anticancer therapy) in the last 6  months | 2 |
| Male patient with uncontrolled hypertension | | Male patient with blood pressure ≥140 mmHg systolic | Male patient with blood pressure ≥140 mmHg systolic | 1 |
| Anemia | | Hemoglobin <12 g/dL in men or <11 g/dL in women | Hemoglobin < 12 g/dL / 7.45 mmol/L in men or <11 g/dL / 6.83 mmol/L in women | 1.5 |
| History of bleeding | | Prior major or non-major clinically relevant bleeding event, rectal  bleeding, frequent nose bleeding or hematuria | Documented history of major or non-major clinically relevant bleeding event, rectal  bleeding, frequent nose bleeding or hematuria | 1.5 |
| Renal dysfunction | | eGFR 30-60 ml/min | eGFR 30-60 ml/min | 1.5 |
| Age | | ≥75 years | ≥75 years | 1.5 |
| **UTAH bleeding risk score**[9] | | | | |
| Age | | >54 years | >54 years | 1 |
| Coronary artery disease | | Not defined | Documented history of coronary artery disease or myocardial infarction | 1 |
| Renal disease | | Chronic kidney disease (not defined) | Either of the following present for ≥3 months: eGFR <60 mL/min, albuminuria (ACR ≥ 30mg/g), urine sediment abnormalities, abnormalities detected by histology, structural abnormalities detected by imaging | 1 |
| Glucose | | > 107 mg/dL / > 5.9 mmol/L | > 107 mg/dL / > 5.9 mmol/L | 1 |
| Mean pulmonary artery pressure (MPAP) | | MPAP < 18 mmHg | MPAP < 18 mmHg | 2 |
| History of bleeding | | History of previous bleeds (not defined) | Any prior documented bleeding event | 2 |
| Right ventricle dysfunction | | Severe dysfunction (not defined) | Poor right ventricle function on cardiac ultrasound as assessed by the performing professional | 1 |
| Abbreviations: ULN: upper limit of normal, AST: aspartate transaminase, ALT: alanine transaminase, AP: alkaline phosphatase, N.A.: not applicable, LVAD: left ventricular assist device, QIO: quality improvement organization, ICD-9-CM: International Classification of Diseases, Ninth Revision, Clinical Modification, eGFR: estimated glomerular filtration rate | | | | |

| **Supplementary Table S4. Approximated cumulative incidence of bleeding (i.e. predicted probability) for each score** | | | |
| --- | --- | --- | --- |
| **Risk score** | **Total score** | **Bleeds (original article)** | **(Approximated) cumulative incidence** |
| **HAS-BLED**[3] | 0 | 1.13 / 100PY | 0.01123639 / year |
|  | 1 | 1.02 / 100PY | 0.01014816 / year |
|  | 2 | 1.88 / 100PY | 0.01862438 / year |
|  | 3 | 3.74 / 100PY | 0.03670926 / year |
|  | 4 | 8.70 / 100PY | 0.08332290 / year |
|  | 5 | 12.50 / 100PY | 0.1175031 / year |
| **ATRIA**[5] | ≤3 | 0.76 / 100PY | 0.007571193 / year |
|  | 4 | 2.62 / 100PY | 0.025859758 / year |
|  | ≥5 | 5.76 / 100PY | 0.055972517 / year |
| **HEMORR_2_HAGES**[4] | 0 | 1.9 / 100PY | 0.05069038 / 1000 days |
|  | 1 | 2.5 / 100PY | 0.06615773 / 1000 days |
|  | 2 | 5.3 / 100PY | 0.13507215 / 1000 days |
|  | 3 | 8.4 / 100PY | 0.20545399 / 1000 days |
|  | 4 | 10.4 / 100PY | 0.24779212 / 1000 days |
|  | ≥5 | 12.3 / 100PY | 0.28592183 / 1000 days |
| **OBRI**[6] | 0 | 3% / year | 0.03 / year |
|  | 1 | 12% / year | 0.12 / year |
|  | 2 | 12% / year | 0.12 / year |
|  | 3 | 48% / year | 0.48 / year |
|  | 4 | 48% / year | 0.48 / year |
| **VTE-BLEED**[7] | 0 | 2.8% (30 days – 6 months) | 0.028 (30 days – 6 months) |
|  | 1 | 2.8% (30 days – 6 months) | 0.028 (30 days – 6 months) |
|  | 1.5 | 2.8% (30 days – 6 months) | 0.028 (30 days – 6 months) |
|  | ≥2 | 12.6% (30 days – 6 months) | 0.126 (30 days – 6 months) |
| **AF-BLEED**[8] | ≤3 | 1.5% / 180 days | 0.015 / 180 days |
|  | >3 | 3.9% / 180 days | 0.039 / 180 days |
| **UBRS**[9] | ≤1 | 4.8% / 3 years | 0.048 / 3 years |
|  | 2 | 39.8% / 3 years | 0.398 / 3 years |
|  | 3 | 39.8% / 3 years | 0.398 / 3 years |
|  | 4 | 39.8% / 3 years | 0.398 / 3 years |
|  | ≥5 | 83.8% / 3 years | 0.838 / 3 years |
| Abbreviations: PY: patient-years, OBRI: Outpatient Bleeding Risk Index, UBRS: Utah Bleeding Risk Score | | | |

**Strategy for converting event rates (EVR) to approximated cumulative incidences (CI)**

To approximate CIs for risk scores that provided EVRs, we applied the following formula:

*CI* = 1 – *e^-IR x T^*.

where *IR* is the incidence rate (equivalent to the EVR) and *T* the timeframe of the derivation cohort, assuming a consistent incidence rate over time. The calculated CIs served as predicted probabilities for major bleeding (MB).

As an illustration, consider the EVR among patients with a HAS-BLED score of 2, which was 1.88 MB / 100 patients years (PY) in the original cohort. Consequently, we assigned patients with a score of 2 a predicted probability of:

1 - *e^-(1.88/100) x 1^* = 0.01862438 MB / year.

The approximated CI per reported EVR for each validated risk scores is detailed in Table S4.

| **Supplementary Table S5. Cumulative incidences of major bleeding and death as competing event** | | | |
| --- | --- | --- | --- |
| **Criteria** | **Timeframe** | **Cumulative incidence MB, % (95%CI)** | **Cumulative incidence death as competing event, % (95%CI)** |
| **ISTH** | 1 month | 26.9 (18.3 – 35.5) | 4.81 (0.67 – 8.94) |
|  | 3 months | 37.5 (28.2 – 46.9) | 6.76 (1.89 – 11.63) |
|  | 6 months | 39.5 (30.0 – 49.0) | 7.74 (2.56 – 12.92) |
|  | 1 years | 50.6 (40.8 – 60.4) | 9.75 (3.96 – 15.54) |
|  | 2 years | 55.9 (46.1 – 65.8) | 13.01 (6.34 – 19.69) |
|  | 3 years | 61.7 (51.9 – 71.4) | 15.42 (8.12 – 22.72) |
|  | 5 years | 71.2 (61.5 – 80.8) | 19.81 (11.33 – 28.29) |
|  | Max. FU (12.4 years) | 75.7 (65.5 – 85.9) | 22.06 (12.56 – 31.56) |
| **INTERMACS** | 1 month | 23.1 (14.9 – 31.2) | 5.77 (1.26 – 10.28) |
|  | 3 months | 31.8 (22.8 – 40.8) | 9.65 (3.93 – 15.36) |
|  | 6 months | 33.7 (24.6 – 42.9) | 11.60 (5.39 – 17.81) |
|  | 1 years | 43.8 (34.1 – 53.5) | 14.62 (7.73 – 21.52) |
|  | 2 years | 47.0 (37.2 – 56.8) | 20.07 (12.11 – 28.04) |
|  | 3 years | 52.8 (42.8 – 62.8) | 22.54 (14.10 – 30.98) |
|  | 5 years | 62.4 (52.2 – 72.7) | 28.48 (18.88 – 38.08) |
|  | Max. FU (12.4 years) | 67.0 (56.0 – 78.0) | 30.75 (20.33 – 41.17) |
| **INTERMACS+** | 1 month | 26.9 (18.3 – 35.5) | 4.81 (0.67 – 8.94) |
|  | 3 months | 37.5 (28.2 – 46.9) | 6.76 (1.89 – 11.63) |
|  | 6 months | 39.5 (30.0 – 49.0) | 7.74 (2.56 – 12.92) |
|  | 1 years | 50.6 (40.8 – 60.4) | 9.75 (3.96 – 15.54) |
|  | 2 years | 55.9 (46.1 – 65.8) | 13.01 (6.34 – 19.69) |
|  | 3 years | 61.7 (51.9 – 71.4) | 15.42 (8.12 – 22.72) |
|  | 5 years | 71.2 (61.5 – 80.8) | 19.81 (11.33 – 28.29) |
|  | Max. FU (12.4 years) | 75.7 (65.5 – 85.9) | 22.06 (12.56 – 31.56) |
| Major bleeding was evaluated according to ISTH (International Society on Thrombosis and Haemostasis), INTERMACS (Interagency Registry for Mechanically Assisted Circulatory Support) and INTERMACS + intracranial bleeding criteria. Abbreviations: MB: major bleeding, Max. FU: maximum follow-up duration | | | |

**
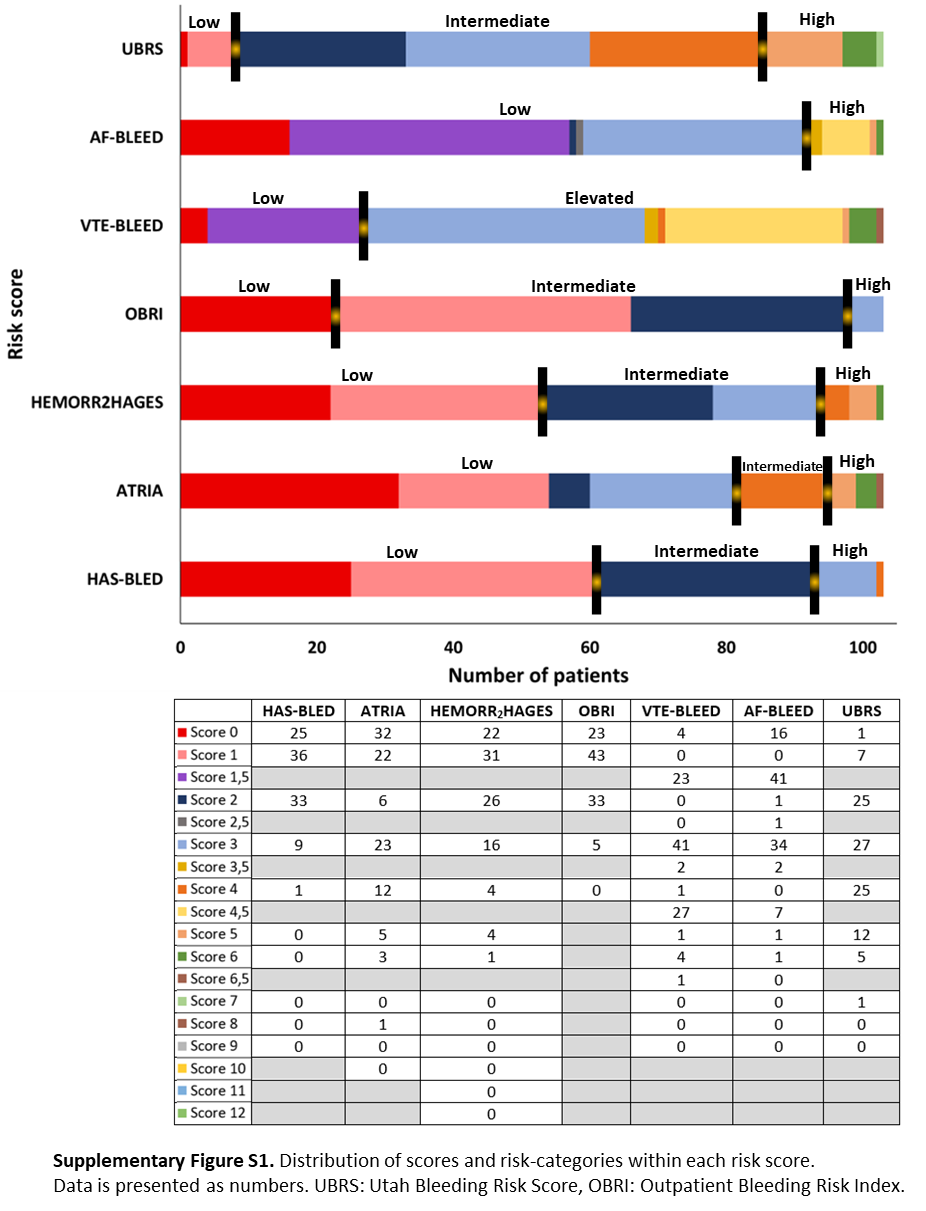
**

| **Supplementary Table S6. Distribution of predictors and comparison with derivation cohorts** | | |
| --- | --- | --- |
| **Predictors in each risk score** | **Distribution of predictors among cohorts** | |
|  | **Development cohort** | **LUMC-LVAD cohort** |
| **HAS-BLED**[3] | | |
| Hypertension | 11.2%^a^ | 0 (0%) |
| Renal disease | 5.3^a^ | 3 (2.9%) |
| Liver disease | N.A. | 2 (1.9%) |
| Stroke history | 5.4%^a^ | 10 (9.6%) |
| Prior major bleeding / predisposition to bleeding | Prior major bleeding: 1.8%^a^ | 50 (48.1%) |
| Labile INR | N.A. | N.A. |
| Age > 65 years | 51.4^a^ | 45 (43.3%) |
| Medication usage predisposing to bleeding | 32.9%^a^ | 13 (12.5%) |
| Alcohol use | 5.4%^a^ | 10 (9.6%) |
| Mean/median score | N.A. | Median: 1 (Q1 – Q3: 1 – 2) |
| **HEMORR_2_HAGES**[4] | | |
| Hepatic or renal disease | 10.3%^a^ | 5 (4.8%) |
| Alcohol abuse | 0.7%^a^ | 5 (4.8%) |
| Malignancy history | 6.2%^a^ | 21 (20.2%) |
| Age > 75 years | 73.8%^a^ | 0 (0%) |
| Reduced platelet count or function | 23.6%^a^ | 38 (36.5%) |
| Rebleeding risk | 19.4%^a^ | 25 (24.0%) |
| Hypertension | 0.5%^a^ | 0 (0%) |
| Anemia | 11.4%^a^ | 44 (42.3%) |
| Genetic factors | N.A. | N.A. |
| Excessive fall risk | 22.5%^a^ | 0 (0%) |
| Stroke history | 30.5%^a^ | 10 (9.6%) |
| Mean/median score | Mean: 2.2^a^ | Median: 1 (Q1 – Q3: 1 – 2) |
| **ATRIA**[5] | | |
| Anemia | 12.4%^a,b^ | 44 (42.3%) |
| Severe renal disease | Dialysis: 0.4%^a,b^  eGFR < 30ml/min: 2.9%^a,b^ | Total: 3 (2.9%)  Dialysis: 1 (1.0%)  eGFR < 30ml/min: 2 (1.9%) |
| Age ≥75 years | 53.5%^a,b^ | 1 (1.0%) |
| History of bleeding | 15.0%^a,b^ | 25 (24.0%) |
| Hypertension | 62.0%^a,b^ | 34 (32.7%) |
| Mean/median score | N.A. | Median: 1 (Q1 – Q3: 0 – 3) |
| **Outpatient Bleeding Risk Index (OBRI)**[6] | | |
| Age ≥65 years | N.A. | 48 (46.2%) |
| History of gastro-intestinal bleeding | 58 (10%) | 9 (8.7%) |
| Stroke history | 66 (12%) | 10 (9.6%) |
| Comorbidities | Renal insufficiency: 100 (18%)  Diabetes: 46 (8%)  Recent MI: 24 (4%)  Hematocrit <30%: 47 (8%) | Total: 57 (54.8%)  Renal insufficiency: 33 (31.7%)  Diabetes: 28 (26.9%)  Recent MI: 2 (1.9%)  Hematocrit <30%: 13 (12.5%) |
| Mean/median score | N.A. | Median: 1 (Q1 – Q3: 1 – 2) |
| **VTE-BLEED**[7] | | |
| Active cancer | 114 (4.4%) | 4 (3.8%) |
| Male patient with uncontrolled hypertension | 480 (19%) | 1 (1.0%) |
| Anemia | 227 (8.9%) | 44 (42.3%) |
| History of bleeding | 123 (4.8%) | 25 (24.0%) |
| Renal dysfunction | 274 (11%) | 69 (66.3%) |
| Age ≥60 years | 41% | 73 (70.2%) |
| Mean/median score | N.A. | Median: 3 (Q1 – Q3: 1.5 – 4.5) |
| **AF-BLEED**[8] | | |
| Active cancer | 1880 (10.4%) | 4 (3.8%) |
| Male patient with uncontrolled hypertension | 3621 (20.1%) | 1 (1.0%) |
| Anemia | 2415 (13.4%) | 44 (42.3%) |
| History of bleeding | 3533 (19.6%) | 25 (24.0%) |
| Renal dysfunction | 6490 (36.0%) | 69 (66.3%) |
| Age ≥75 years | 7205 (39.9%) | 1 (1.0%) |
| Mean/median score | N.A. | Median: 1.5 (Q1 – Q3: 1.5 – 3) |
| **Utah bleeding risk score (UBRS)**[9] | | |
| Age >54 years | N.A. | 88 (84.6%) |
| Coronary artery disease | 181 (52%) | 70 (67.3%) |
| Renal disease | 126 (36%) | 55 (52.9%) |
| Glucose > 107 mg/dL / > 5.9 mmol/L | N.A. | 48 (46.2%) |
| MPAP < 18mmHg | N.A. | 12 (11.5%) |
| History of bleeding | 30 (9%) | 25 (24.0%) |
| Right ventricle dysfunction | 52 (15%) | 4 (3.8%) |
| Mean/median score | N.A. | Median: 3 (Q1 – Q3: 2 – 4) |
| ^a^ Baselines in this study were presented as percentages in two or more subgroups. Number and percentage of the total group were estimated by calculating a weighted mean of these percentages.  ^b^ % person-years  Abbreviations: LVAD: left ventricular assist device, N.A.: not applicable, INR: international normalized ratio, Q1 – Q3: quartile 1 – quartile 3, eGFR: estimated glomerular filtration rate, MI: myocardial infarction, MPAP: mean pulmonary artery pressure | | |

| **Supplementary Table S7. Calibration measurements of each risk score to predict major bleeding on their intended timeframe with mortality as competing event** | | | | |
| --- | --- | --- | --- | --- |
| **Criteria** | **Model** | **O/E ratio (95%CI)** | **Intercept (95%CI)** | **Slope (95%CI)** |
| **ISTH** | HAS-BLED | 31.42 (25.94– 38.06) | 3.86 (3.56 – 4.17) | 0.31 (-0.28 – 0.90) |
|  | HEMORR_2_HAGES | 5.18 (4.43 – 6.06) | 2.26 (1.93 – 2.59) | 0.05 (-0.38 – 0.47) |
|  | ATRIA | 36.48 (30.11 – 44.19) | 4.38 (4.05 – 4.71) | -0.03 (-0.46 – 0.40) |
|  | OBRI | 4.31 (3.56 – 5.22) | 1.82 (1.50 – 2.13) | 0.10 (-0.29 – 0.48) |
|  | VTE-BLEED | 1.90 (1.16 – 3.11) | 0.49 (-0.11 – 1.09) | -0.03 (-0.79 – 0.73) |
|  | AF-BLEED | 21.97 (17.23 – 28.01) | 3.33 (2.99 – 3.66) | 0.25 (-0.73 – 1.24) |
|  | UBRS | 1.38 (1.18 – 1.61) | 0.53 (0.23 – 0.84) | 0.14 (-0.18 – 0.46) |
|  |  |  |  |  |
| **INTERMACS** | HAS-BLED | 27.19 (21.82 – 33.87) | 3.61 (3.28 – 3.93) | 0.31 (-0.31 – 0.94) |
|  | HEMORR_2_HAGES | 4.43 (3.68 – 5.35) | 1.87 (1.53 – 2.21) | 0.11 (-0.35 – 0.57) |
|  | ATRIA | 31.56 (25.34 – 39.32) | 4.04 (3.67 – 4.42) | -0.11 (-0.59 – 0.37) |
|  | OBRI | 3.73 (2.99 – 4.64) | 1.55 (1.22 – 1.89) | 0.09 (-0.33 – 0.51) |
|  | VTE-BLEED | 1.42 (0.80 – 2.53) | 0.18 (-0.50 – 0.87) | 0.01 (-0.86 – 0.88) |
|  | AF-BLEED | 18.67 (14.17 – 24.59) | 3.11 (2.76 – 3.47) | 0.50 (-0.50 – 1.49) |
|  | UBRS | 1.18 (0.98 – 1.42) | 0.21 (-0.11 – 0.54) | 0.19 (-0.17 – 0.54) |
|  |  |  |  |  |
| **INTERMACS+** | HAS-BLED | 31.42 (25.94– 38.06) | 3.86 (3.56 – 4.17) | 0.31 (-0.28 – 0.90) |
|  | HEMORR_2_HAGES | 5.18 (4.43 – 6.06) | 2.26 (1.93 – 2.59) | 0.05 (-0.38 – 0.47) |
|  | ATRIA | 36.48 (30.11 – 44.19) | 4.38 (4.05 – 4.71) | -0.03 (-0.46 – 0.40) |
|  | OBRI | 4.31 (3.56 – 5.22) | 1.82 (1.50 – 2.13) | 0.10 (-0.29 – 0.48) |
|  | VTE-BLEED | 1.90 (1.16 – 3.11) | 0.49 (-0.11 – 1.09) | -0.03 (-0.79 – 0.73) |
|  | AF-BLEED | 21.97 (17.23 – 28.01) | 3.33 (2.99 – 3.66) | 0.25 (-0.73 – 1.24) |
|  | UBRS | 1.38 (1.18 – 1.61) | 0.53 (0.23 – 0.84) | 0.14 (-0.18 – 0.46) |
| Major bleeding was evaluated according to ISTH (International Society on Thrombosis and Haemostasis), INTERMACS (Interagency Registry for Mechanically Assisted Circulatory Support) and INTERMACS + intracranial bleeding criteria. Abbreviations: O/E (observed/expected), 95%CI: 95% confidence interval, OBRI: Outpatient Bleeding Risk Score, UBRS: Utah Bleeding Risk Score. | | | | |

| **Supplementary Table S8. Cumulative incidence of MB among HeartWare as compared to HeartMate-3 patients** | | | | |
| --- | --- | --- | --- | --- |
| **Criteria** | **Timeframe** | **HeartWare, cumulative incidence MB, % (95%CI)**  **(n = 94)** | **HeartMate-3, cumulative incidence MB, % (95%CI)**  **(n = 10)** | **Gray’s *p*** |
| **ISTH** | 1 month | 29.0 (19.7 – 38.3) | 10.0 (0.0 – 29.6) | 0.1842 |
|  | 3 months | 38.7 (28.7 – 48.7) | 30.0 (0.0 – 60.4) | 0.4704 |
|  | 6 months | 40.9 (30.8 – 50.9) | 30.0 (0.0 – 60.4) | 0.4187 |
|  | 1 years | 51.6 (41.4 – 61.9) | 45.0 (6.8 – 83.2) | 0.4641 |
|  | 2 years | 57.0 (46.8 – 67.2) | N.A. |  |
|  | 3 years | 62.5 (52.6 – 72.5) | N.A. |  |
|  | 5 years | 71.8 (62.0 – 81.5) | N.A. |  |
|  | Max. FU (12.4 years) | 76.1 (65.9 – 86.3) | N.A. |  |
| **INTERMACS** | 1 month | 26.9 (17.8 – 36.0) | 0.0 (0.0 – 0.0) | 0.0647 |
|  | 3 months | 33.3 (23.7 – 43.0) | 20.0 (0.0 – 46.4) | 0.3172 |
|  | 6 months | 35.5 (25.7 – 45.3) | 20.0 (0.0 – 46.4) | 0.2751 |
|  | 1 years | 46.2 (36.0 – 56.5) | 20.0 (0.0 – 46.4) | 0.1396 |
|  | 2 years | 49.5 (39.2 – 59.7) | N.A. |  |
|  | 3 years | 55.0 (44.7 – 65.2) | N.A. |  |
|  | 5 years | 64.1 (53.8 – 74.4) | N.A. |  |
|  | Max. FU (12.4 years) | 68.4 (57.5 – 79.3) | N.A. |  |
| **INTERMACS+** | 1 month | 29.0 (19.7 – 38.3) | 10.0 (0.0 – 29.6) | 0.1842 |
|  | 3 months | 38.7 (28.7 – 48.7) | 30.0 (0.0 – 60.4) | 0.4704 |
|  | 6 months | 40.9 (30.8 – 50.9) | 30.0 (0.0 – 60.4) | 0.4187 |
|  | 1 years | 51.6 (41.4 – 61.9) | 45.0 (6.8 – 83.2) | 0.4641 |
|  | 2 years | 57.0 (46.8 – 67.2) | N.A. |  |
|  | 3 years | 62.5 (52.6 – 72.5) | N.A. |  |
|  | 5 years | 71.8 (62.0 – 81.5) | N.A. |  |
|  | Max. FU (12.4 years) | 76.1 (65.9 – 86.3) | N.A. |  |
| Major bleeding was evaluated according to ISTH (International Society on Thrombosis and Haemostasis), INTERMACS (Interagency Registry for Mechanically Assisted Circulatory Support) and INTERMACS + intracranial bleeding criteria, with mortality as competing event. Abbreviations: MB: major bleeding, Max. FU: maximum follow-up duration | | | | |

| **Supplementary Table S9. Cumulative incidences of MB within patients initially prescribed clopidogrel vs ASA** | | | | |
| --- | --- | --- | --- | --- |
| **Criteria** | **Timeframe** | **Clopidogrel - cumulative incidence MB, % (95%CI)**  **(n = 83)** | **ASA - cumulative incidence MB, % (95%CI)**  **(n = 11)** | **Gray’s *p*** |
| **ISTH** | 1 month | 28.0 (18.3 – 37.8) | 9.1 (0.0 – 26.9) | 0.1668 |
|  | 3 months | 37.8 (27.2 – 48.4) | 27.3 (0.0 – 55.2) | 0.4028 |
|  | 6 months | 40.2 (29.5 – 50.9) | 27.3 (0.0 – 55.2) | 0.3462 |
|  | 1 years | 52.4 (41.5 – 63.4) | 40.0 (5.9 – 74.1) | 0.3261 |
|  | 2 years | 58.5 (47.8 – 69.3) | 40.0 (5.9 – 74.1) | 0.2748 |
|  | 3 years | 64.7 (54.2 – 75.2) | N.A. | N.A. |
|  | 5 years | 74.7 (64.7 – 84.7) | N.A. | N.A. |
|  | Max. FU (12.4 years) | 79.5 (69.0– 90.0) | N.A. | N.A. |
| **INTERMACS** | 1 month | 26.8 (17.2 – 36.5) | 0.0 (0.0 – 0.0) | 0.0538 |
|  | 3 months | 34.1 (23.8 – 44.5) | 18.2 (0.0 – 42.3) | 0.2353 |
|  | 6 months | 36.6 (26.1 – 47.1) | 18.2 (0.0 – 42.3) | 0.1956 |
|  | 1 years | 48.8 (37.9 – 59.7) | 18.2 (0.0 – 42.3) | 0.0781 |
|  | 2 years | 52.4 (41.5 – 63.4) | 18.2 (0.0 – 42.3) | 0.0651 |
|  | 3 years | 58.5 (47.7 – 69.3) | N.A. | N.A. |
|  | 5 years | 68.5 (57.9 – 79.1) | N.A. | N.A. |
|  | Max. FU (12.4 years) | 73.2 (62.0 – 84.4) | N.A. | N.A. |
| **INTERMACS+** | 1 month | 29.0 (19.7 – 38.3) | 9.1 (0.0 – 26.9) | 0.1668 |
|  | 3 months | 38.7 (28.7 – 48.7) | 27.3 (0.0 – 55.2) | 0.4028 |
|  | 6 months | 40.9 (30.8 – 50.9) | 27.3 (0.0 – 55.2) | 0.3462 |
|  | 1 years | 51.6 (41.4 – 61.9) | 40.0 (5.9 – 74.1) | 0.3261 |
|  | 2 years | 57.0 (46.8 – 67.2) | 40.0 (5.9 – 74.1) | 0.2748 |
|  | 3 years | 62.5 (52.6 – 72.5) | N.A. | N.A. |
|  | 5 years | 71.8 (62.0 – 81.5) | N.A. | N.A. |
|  | Max. FU (12.4 years) | 76.1 (65.9 – 86.3) | N.A. | N.A. |
| Major bleeding was evaluated according to ISTH (International Society on Thrombosis and Haemostasis), INTERMACS (Interagency Registry for Mechanically Assisted Circulatory Support) and INTERMACS + intracranial bleeding criteria, with mortality as competing event. Abbreviations: MB: major bleeding, ASA: acetylsalicylic acid or carbasalate calcium**,** Max. FU: maximum follow-up duration | | | | |

| **Supplementary Table S10. Clinical characteristics of the entire cohort vs the complete case cohort** | | |
| --- | --- | --- |
| **Characteristics** | **All (n = 104)** | **Complete case (n = 94)** |
| **Age at implantation in years**, median (Q1 - Q3) | 64 (58 - 68) | 64 (58.3 – 68) |
| **Women**, n (%) | 21 (20.2) | 19 (20.2) |
| **Heart failure etiology,** n (%)  Ischemic cardiomyopathy  Non-ischemic dilative cardiomyopathy  Valvular heart disease  Congenital heart disease  Hypertrophic cardiomyopathy | 58 (55.8)  35 (33.7)  6 (5.8)  4 (3.8)  1 (1.0) | 50 (53.2)  34 (36.2)  5 (5.3)  4 (4.3)  1 (1.1) |
| **NYHA class**, n (%)  III  IV | 48 (46.2)  56 (53.8) | 44 (46.8)  50 (53.2) |
| **INTERMACS profile pre-implantation,** n (%)  Profile 1  Profile 2  Profile 3  Profile 4  Profile 5 | 4 (3.8)  18 (17.3)  42 (40.4)  28 (26.9)  12 (11.5) | 3 (3.2)  16 (17.0)  39 (41.5)  26 (27.7)  10 (10.6) |
| **Risk scores pre-implantation**, median (Q1 – Q3)  HAS-BLED  HEMORR_2_HAGES  ATRIA  OBRI  VTE-BLEED  AF-BLEED  UBRS | 1 (1 – 2)  1 (1 – 2)  1 (0 – 3)  1 (1 – 2)  3 (1.5 – 4.5)  1.5 (1.5 – 3.0)  3 (2 – 4) | 1 (1 – 2)  1 ( 1 – 2)  1 (0 – 3)  1 (1 – 2)  3 (1.5 – 4.5)  1.5 (1.5 – 3.0)  3 (2 – 4) |
| **LVAD device implanted**, n (%)  HeartWare  HeartMate-3 | 94 (90.4)  10 (9.6) | 84 (89.4)  10 (10.6) |
| **Device strategy**, n (%)  Destination therapy  Rescue therapy  Bridge to recovery | 101 (97.1)  2 (1.9)  1 (1.0) | 92 (97.9)  1 (1.1)  1 (1.1) |
| Abbreviations: n: number, Q1 – Q3: quartile 1 – quartile 3, NYHA: New York Heart Association, INTERMACS: Interagency Registry for Mechanically Assisted Circulatory Support, OBRI: Outpatient Bleeding Risk Index, UBRS: Utah Bleeding Risk Score, LVAD: left ventricular assist device. | | |


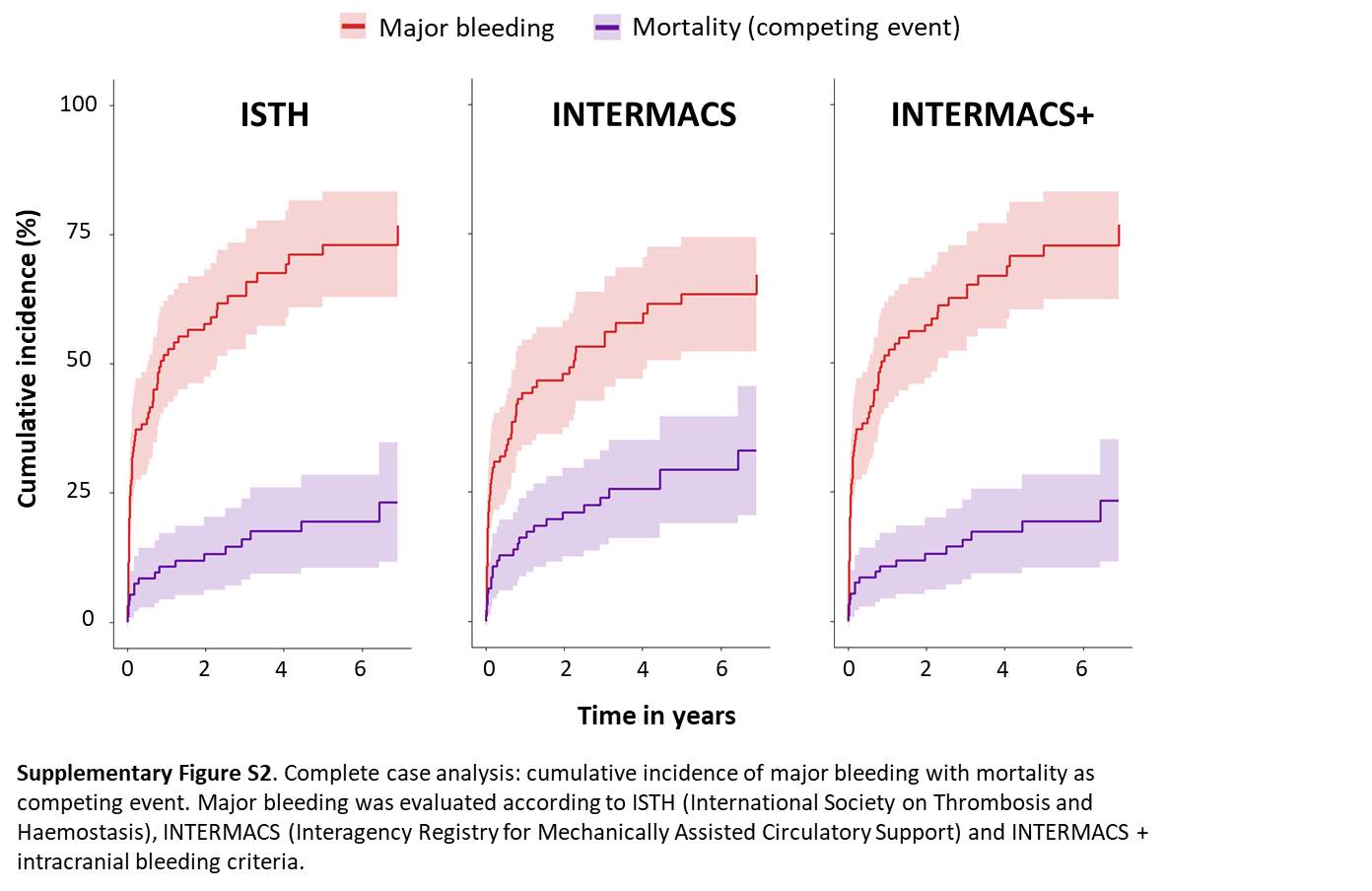


| **Supplementary Table S11. Discriminative ability of the risk scores for predicting major bleeding on their intended timeframe - complete-case analysis** | | | | |
| --- | --- | --- | --- | --- |
| **Score** | **Timeframe** | **AUC_t_ (95%CI)** | | |
|  |  | **ISTH** | **INTERMACS** | **INTERMACS+** |
| HAS-BLED | 1 year | 0.55 (0.44 – 0.67) | 0.53 (0.41 – 0.65) | 0.55 (0.44 – 0.67) |
| HEMORR_2_HAGES | 1000 days | 0.50 (0.38 – 0.63) | 0.52 (0.40 – 0.64) | 0.50 (0.38 – 0.63) |
| ATRIA | 1 year | 0.46 (0.37 – 0.54) | 0.45 (0.36 – 0.53) | 0.46 (0.37 – 0.54) |
| OBRI | 1 year | 0.54 (0.45 – 0.63) | 0.53 (0.44 – 0.63) | 0.54 (0.45 – 0.63) |
| VTE-BLEED | 30 days – 6 months | 0.49 (0.34 – 0.64) | 0.50 (0.33 – 0.66) | 0.49 (0.34 – 0.64) |
| AF-BLEED | 180 days | 0.52 (0.46 – 0.58) | 0.53 (0.47 – 0.60) | 0.52 (0.46 – 0.58) |
| UBRS | 3 year | 0.60 (0.51 – 0.68) | 0.59 (0.50 – 0.68) | 0.60 (0.51 – 0.68) |
| Abbreviations: AUC_t_: cumulative area under the curve, 95%CI: 95% confidence interval, OBRI: Outpatient Bleeding Risk Index, UBRS: Utah Bleeding Risk Score. Major bleeding was evaluated according to ISTH (International Society on Thrombosis and Haemostasis), INTERMACS (Interagency Registry for Mechanically Assisted Circulatory Support) and INTERMACS + intracranial bleeding criteria with mortality as competing event. | | | | |


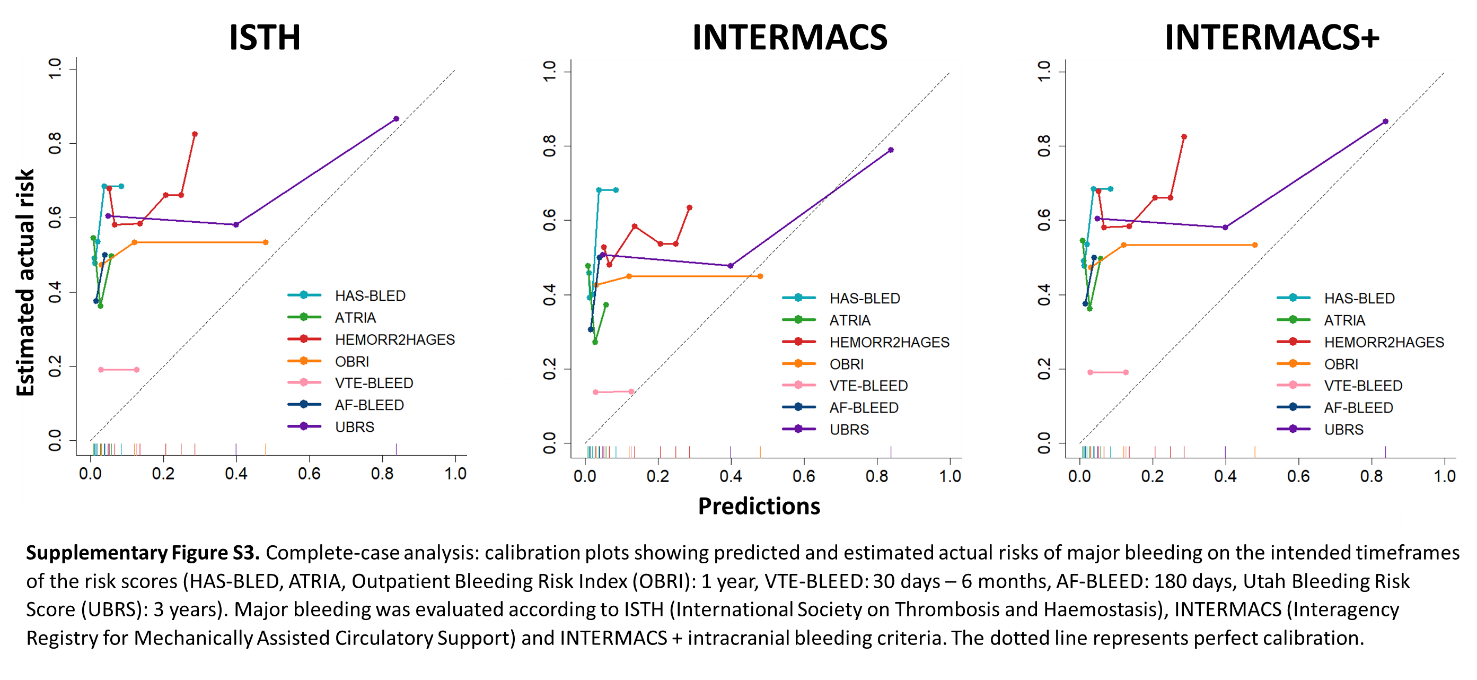


| **Supplementary Table S12. Discriminative ability of the risk scores for predicting major bleeding on their intended timeframe - non-competing risk analysis** | | | | |
| --- | --- | --- | --- | --- |
| **Score** | **Timeframe** | **AUC_t_ (95%CI)** | | |
|  |  | **ISTH** | **INTERMACS** | **INTERMACS+** |
| HAS-BLED | 1 year | 0.58 (0.47 – 0.69) | 0.56 (0.45 – 0.68) | 0.58 (0.47 – 0.69) |
| HEMORR_2_HAGES | 1000 days | 0.49 (0.35 – 0.64) | 0.52 (0.38 – 0.67) | 0.49 (0.35 – 0.64) |
| ATRIA | 1 year | 0.50 (0.41 – 0.58) | 0.48 (0.40 – 0.57) | 0.50 (0.41 – 0.58) |
| OBRI | 1 year | 0.56 (0.46 – 0.65) | 0.55 (0.45 – 0.65) | 0.56 (0.46 – 0.65) |
| VTE-BLEED | 30days – 6 months | 0.50 (0.36 – 0.64) | 0.51 (0.35 – 0.66) | 0.50 (0.36 – 0.64) |
| AF-BLEED | 180d | 0.51 (0.44 – 0.57) | 0.52 (0.45 – 0.59) | 0.51 (0.44 – 0.57) |
| UBRS | 3y | 0.58 (0.47 – 0.69) | 0.58 (0.47 – 0.69) | 0.58 (0.47 – 0.69) |
| Death without having experienced a major bleeding was considered a censoring event.  Abbreviations: AUC_t_: cumulative area under the curve, 95%CI: 95% confidence interval, OBRI: Outpatient Bleeding Risk Index, UBRS: Utah Bleeding Risk Score. Major bleeding was evaluated according to ISTH (International Society on Thrombosis and Haemostasis), INTERMACS (Interagency Registry for Mechanically Assisted Circulatory Support) and INTERMACS + intracranial bleeding criteria. | | | | |

**
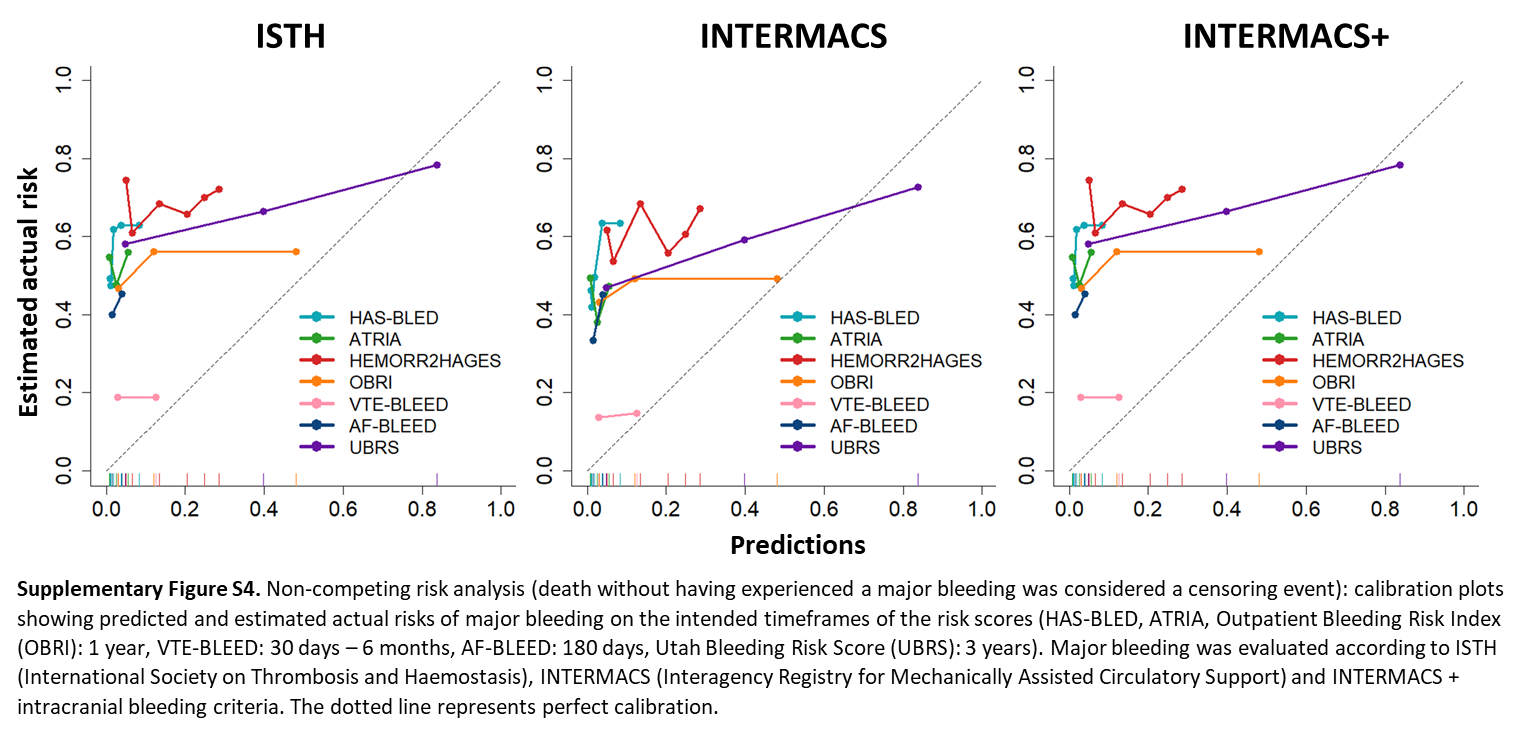
**

| **Supplementary Table S13. Discriminative ability (according to Harrell *C*-index) of the risk scores for predicting major bleeding on their intended timeframe** | | | | |
| --- | --- | --- | --- | --- |
| **Score** | **Timeframe** | **Harrell *C*-index (95%CI)** | | |
|  |  | **ISTH** | **INTERMACS** | **INTERMACS+** |
| HAS-BLED | 1 year | 0.51 (0.43 – 0.58) | 0.50 (0.42 – 0.58) | 0.51 (0.43 – 0.58) |
| HEMORR_2_HAGES | 1000 days | 0.51 (0.44 – 0.57) | 0.53 (0.46 – 0.61) | 0.51 (0.44 – 0.57) |
| ATRIA | 1 year | 0.50 (0.44 – 0.56) | 0.49 (0.43 – 0.55) | 0.50 (0.44 – 0.56) |
| OBRI | 1 year | 0.51 (0.44 – 0.58) | 0.50 (0.44 – 0.57) | 0.51 (0.44 – 0.58) |
| VTE-BLEED | 30 days – 6 months | 0.51 (0.44 – 0.56) | 0.50 (0.33 – 0.64) | 0.51 (0.44 – 0.56) |
| AF-BLEED | 180 days | 0.52 (0.46 – 0.57) | 0.53 (0.47 – 0.60) | 0.52 (0.46 – 0.57) |
| UBRS | 3 years | 0.55 (0.48 – 0.61) | 0.56 (0.48 – 0.63) | 0.55 (0.48 – 0.61) |
| Abbreviations: 95% confidence interval, OBRI: Outpatient Bleeding Risk Index, UBRS: Utah Bleeding Risk Score. Major bleeding was evaluated according to ISTH (International Society on Thrombosis and Haemostasis), INTERMACS (Interagency Registry for Mechanically Assisted Circulatory Support) and INTERMACS + intracranial bleeding criteria with mortality as competing event. | | | | |

| **Supplementary Table S14. Types of major bleeding, according to ISTH, INTERMACS+ and INTERMACS criteria, beyond 14 days post-LVAD implantation with death and thrombolysis as competing events** | |
| --- | --- |
| **ISTH and INTERMACS+** | **INTERMACS** |
| Gastrointestinal: 23  Intracranial: 14  Driveline exit site: 3  (Sub)cutaneous / intramuscular: 3  Mediastinal: 3  Epistaxis: 3  Pleural space: 2  Implantable cardioverter-defibrillator pocket: 1  Intraabdominal: 1  Retroperitoneal: 1  Pulmonary: 1  Intraoral: 1  Fatal hemorrhagic shock of unknown etiology: 1 | Gastrointestinal: 24  Epistaxis: 4  Driveline exit site: 3  Mediastinal: 3  (Sub)cutaneous / intramuscular: 3  Pleural space: 2  Implantable cardioverter-defibrillator pocket: 1  Intraabdominal: 1  Retroperitoneal: 1  Pulmonary: 1  Fatal hemorrhagic shock of unknown etiology: 1  Intraoral: 1 |

**
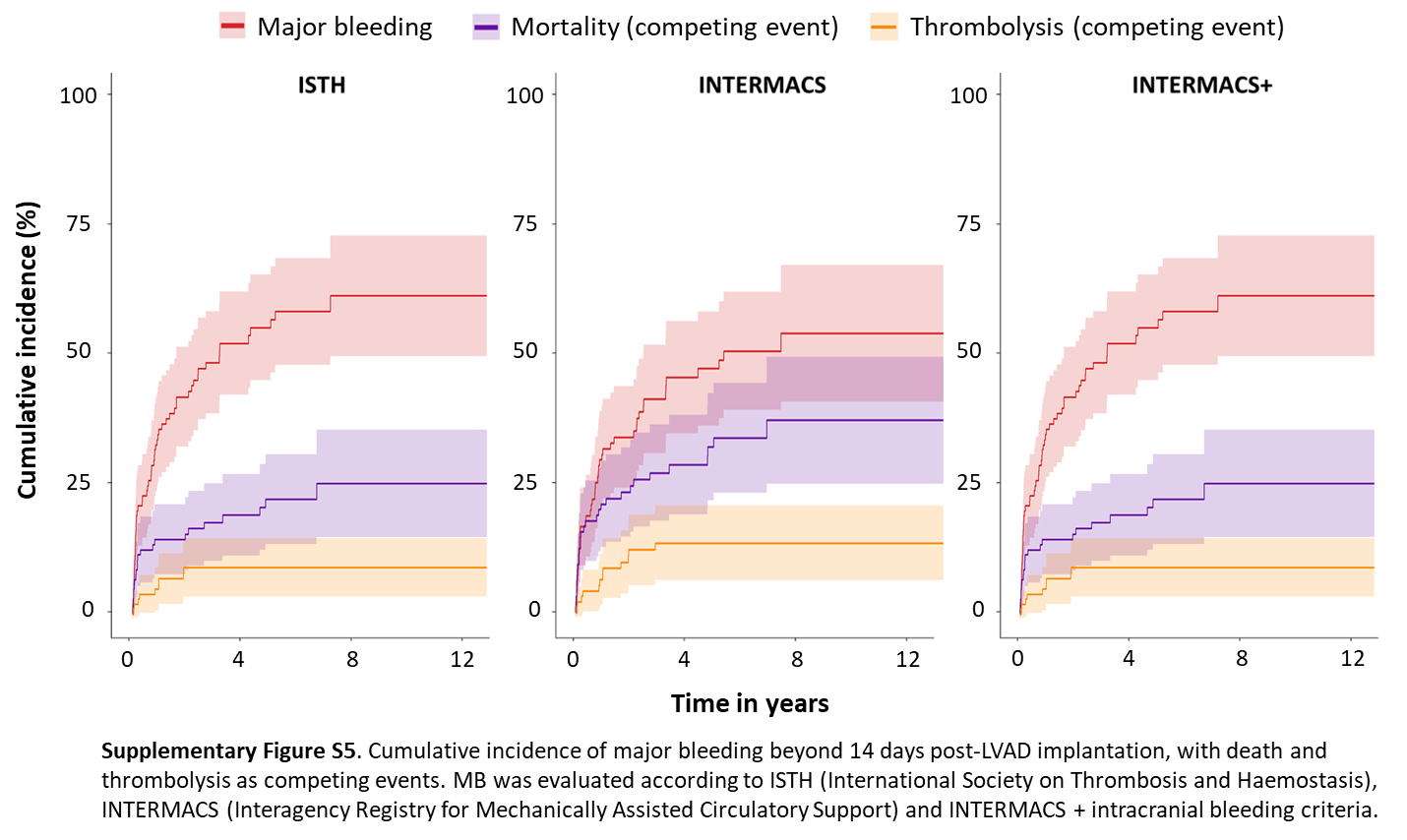
**

| **Supplementary Table S15. Discriminative ability of the risk scores for predicting major bleeding on their intended timeframe – only including major bleeding beyond 14 days post-LVAD implantation** | | | | |
| --- | --- | --- | --- | --- |
| **Score** | **Timeframe** | **AUC_t_ (95%CI)** | | |
|  |  | **ISTH** | **INTERMACS** | **INTERMACS+** |
| HAS-BLED | 1 year | 0.56 (0.45 – 0.68) | 0.53 (0.41 – 0.66) | 0.56 (0.45 – 0.68) |
| HEMORR_2_HAGES | 1000 days | 0.48 (0.36 – 0.60) | 0.46 (0.34 – 0.59) | 0.48 (0.36 – 0.60) |
| ATRIA | 1 year | 0.49 (0.40 – 0.57) | 0.47 (0.39 – 0.55) | 0.49 (0.40 – 0.57) |
| OBRI | 1 year | 0.55 (0.46 – 0.64) | 0.53 (0.43 – 0.63) | 0.55 (0.46 – 0.64) |
| VTE-BLEED | 30 days – 6 months | 0.49 (0.36 – 0.62) | 0.50 (0.36 – 0.65) | 0.49 (0.36 – 0.62) |
| AF-BLEED | 180 days | 0.48 (0.42 – 0.55) | 0.50 (0.42 – 0.58) | 0.48 (0.42 – 0.55) |
| UBRS | 3 year | 0.52 (0.43 – 0.61) | 0.49 (0.40 – 0.58) | 0.52 (0.43 – 0.61) |
| Abbreviations: AUC_t_: cumulative area under the curve, 95%CI: 95% confidence interval, OBRI: Outpatient Bleeding Risk Index, UBRS: Utah Bleeding Risk Score. Major bleeding was evaluated according to ISTH (International Society on Thrombosis and Haemostasis), INTERMACS (Interagency Registry for Mechanically Assisted Circulatory Support) and INTERMACS + intracranial bleeding criteria with mortality and thrombolysis as competing events | | | | |

**
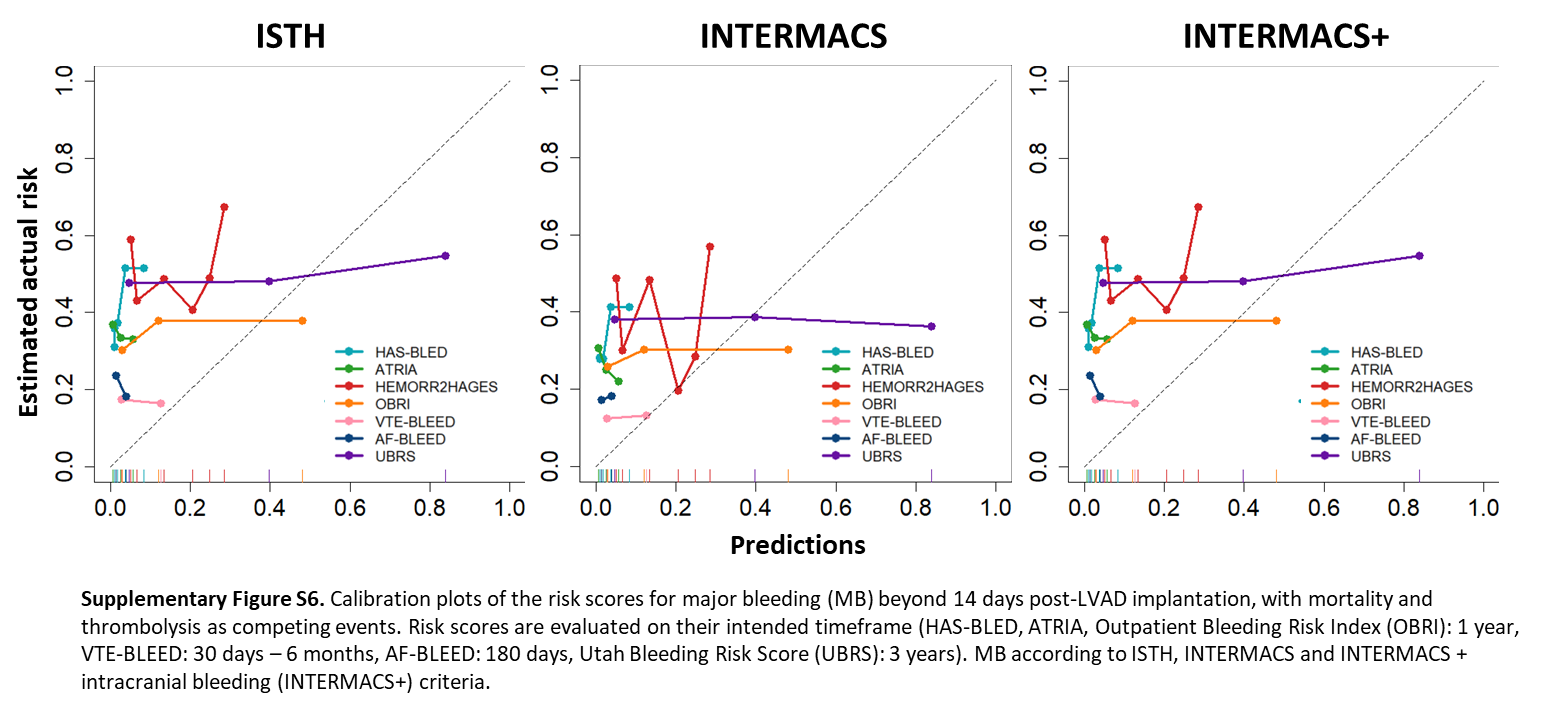
**

| **Supplementary Table S16. Association between risk scores and major bleeding outcomes over the maximum follow-up duration** | | | |
| --- | --- | --- | --- |
|  | **Subdistribution hazard ratio (95%CI)** | | |
|  | **ISTH** | **INTERMACS** | **INTERMACS+** |
| **HAS-BLED** Discrete score | 0.95 (0.75 – 1.21) | 1.00 (0.77 – 1.30) | 0.95 (0.75 – 1.21) |
| Low risk  Intermediate risk  High risk | Ref  0.85 (0.49 – 1.45)  1.10 (0.50 – 2.41) | Ref  0.72 (0.40 – 1.30)  1.32 (0.60 – 2.87) | Ref  0.85 (0.49 – 1.45)  1.10 (0.50 – 2.41) |
| **ATRIA**  Discrete score | 1.00 (0.89 – 1.13) | 1.01 (0.90 – 1.15) | 1.00 (0.89 – 1.13) |
| Low risk  Intermediate risk  High risk | Ref  0.70 (0.32 – 1.55)  1.30 (0.62 – 2.72) | Ref  0.69 (0.30 – 1.61)  1.19 (0.53 – 2.69) | Ref  0.70 (0.32 – 1.55)  1.30 (0.62 – 2.72) |
| **HEMORR_2_HAGES** Discrete score | 1.02 (0.87 – 1.20) | 1.02 (0.87 – 1.21) | 1.02 (0.87 – 1.20) |
| Low risk  Intermediate risk  High risk | Ref  1.14 (0.71 – 1.83)  0.97 (0.39 – 2.39) | Ref  1.29 (0.77 – 2.14)  0.94 (0.36 – 2.49) | Ref  1.14 (0.71 – 1.83)  0.97 (0.39 – 2.39) |
| **OBRI** Discrete score | 0.92 (0.70 – 1.21) | 0.94 (0.70 – 1.27) | 0.92 (0.70 – 1.21) |
| Low risk  Intermediate risk  High risk | Ref  0.83 (0.50 – 1.39)  1.03 (0.36 – 2.92) | Ref  0.89 (0.50 – 1.58)  1.36 (0.48 – 3.90) | Ref  0.83 (0.50 – 1.39)  1.03 (0.36 – 2.92) |
| **VTE-BLEED**  Discrete score | 0.95 (0.82 – 1.09) | 0.99 (0.84 – 1.16) | 0.95 (0.82 – 1.09) |
| Low risk  Elevated risk | Ref  0.79 (0.51 – 1.24) | Ref  0.95 (0.57 – 1.57) | Ref  0.79 (0.51 – 1.24) |
| **AF-BLEED**  Discrete score | 1.08 (0.91 – 1.28) | 1.11 (0.92 – 1.34) | 1.08 (0.91 – 1.28) |
| Low risk  High risk | Ref  1.44 (0.69 – 3.00) | Ref  1.49 (0.67 – 3.33) | Ref  1.44 (0.69 – 3.00) |
| **UBRS**  Discrete score | 1.00 (0.83 – 1.22) | 1.03 (0.83 – 1.29) | 1.00 (0.83 – 1.22) |
| Low risk  Intermediate risk  High risk | Ref  0.69 (0.35 – 1.35)  1.43 (0.62 – 3.26) | Ref  0.87 (0.37 – 2.06)  1.97 (0.73 – 5.36) | Ref  0.69 (0.35 – 1.35)  1.43 (0.62 – 3.26) |
| Univariate Fine-Gray model was used to obtain subdistribution hazard ratio with 95%CI. The risk scores were evaluated as discrete variables and as a categorical variables (e.g. low, intermediate and high risk). Major bleeding was evaluated according to ISTH (International Society on Thrombosis and Haemostasis), INTERMACS (Interagency Registry for Mechanically Assisted Circulatory Support) and INTERMACS + intracranial bleeding criteria. Abbreviations: OBRI: Outpatient Bleeding Risk Index, UBRS: Utah Bleeding Risk Score. | | | |

**REFERENCES**

1. Kaatz, S., et al., *Definition of clinically relevant non-major bleeding in studies of anticoagulants in atrial fibrillation and venous thromboembolic disease in non-surgical patients: communication from the SSC of the ISTH.* J Thromb Haemost, 2015. **13**(11): p. 2119-26.

2. Kormos, R.L., et al., *Updated definitions of adverse events for trials and registries of mechanical circulatory support: A consensus statement of the mechanical circulatory support academic research consortium.* J Heart Lung Transplant, 2020. **39**(8): p. 735-750.

3. Pisters, R., et al., *A novel user-friendly score (HAS-BLED) to assess 1-year risk of major bleeding in patients with atrial fibrillation: the Euro Heart Survey.* Chest, 2010. **138**(5): p. 1093-100.

4. Gage, B.F., et al., *Clinical classification schemes for predicting hemorrhage: results from the National Registry of Atrial Fibrillation (NRAF).* Am Heart J, 2006. **151**(3): p. 713-9.

5. Fang, M.C., et al., *A new risk scheme to predict warfarin-associated hemorrhage: The ATRIA (Anticoagulation and Risk Factors in Atrial Fibrillation) Study.* J Am Coll Cardiol, 2011. **58**(4): p. 395-401.

6. Beyth, R.J., L.M. Quinn, and C.S. Landefeld, *Prospective evaluation of an index for predicting the risk of major bleeding in outpatients treated with warfarin.* Am J Med, 1998. **105**(2): p. 91-9.

7. Klok, F.A., et al., *Prediction of bleeding events in patients with venous thromboembolism on stable anticoagulation treatment.* Eur Respir J, 2016. **48**(5): p. 1369-1376.

8. Chu, G., et al., *Tailoring anticoagulant treatment of patients with atrial fibrillation using a novel bleeding risk score.* Heart, 2020.

9. Yin, M.Y., et al., *Novel Model to Predict Gastrointestinal Bleeding During Left Ventricular Assist Device Support.* Circ Heart Fail, 2018. **11**(11): p. e005267.
